# Logical-rules and the classification of integral dimensions: individual differences in the processing of arbitrary dimensions

**DOI:** 10.3389/fpsyg.2014.01531

**Published:** 2015-01-09

**Authors:** Anthea G. Blunden, Tony Wang, David W. Griffiths, Daniel R. Little

**Affiliations:** Melbourne School of Psychological Sciences, The University of MelbourneMelbourne, VIC, Australia

**Keywords:** integrality, separability, serial vs. parallel, coactivation, holistic processing, categorization, computational modeling, reaction time

## Abstract

A variety of converging operations demonstrate key differences between separable dimensions, which can be analyzed independently, and integral dimensions, which are processed in a non-analytic fashion. A recent investigation of response time distributions, applying a set of logical rule-based models, demonstrated that integral dimensions are pooled into a single coactive processing channel, in contrast to separable dimensions, which are processed in multiple, independent processing channels. This paper examines the claim that arbitrary dimensions created by factorially morphing four faces are processed in an integral manner. In two experiments, 16 participants completed a categorization task in which either upright or inverted morph stimuli were classified in a speeded fashion. Analyses focused on contrasting different assumptions about the psychological representation of the stimuli, perceptual and decisional separability, and the processing architecture. We report consistent individual differences which demonstrate a mixture of some observers who demonstrate coactive processing with other observers who process the dimensions in a parallel self-terminating manner.

## Introduction

Understanding how our perceptual systems process multidimensional stimuli provides fundamental insights into basic cognitive operations such as categorization (Ashby and Gott, 1988; Fifić et al., 2010; Little et al., 2011), object representation (Folstein et al., 2013), and recognition memory (Nosofsky et al., 2011, 2012). Of critical importance is the difference between stimuli that consist of either separable or integral perceptual dimensions. Separable dimensions are those which can be attended to and analyzed in isolation, such as size and shape (Attneave, 1950; Torgenson, 1958; Shepard, 1964; Garner, 1974, 1978). In contrast, integral dimensions are thought to be psychologically “fused,” such that one integral dimension cannot be attended to at the expense of the other; both must be processed together (Garner, 1974; Burns and Shepp, 1988).

Although many stimulus dimensions have been studied in the information processing literature, research demonstrating the integrality of stimulus dimensions has focused primarily on the dimensions of brightness and saturation of Munsell colors for visual stimuli (Shepard and Chang, 1963; Garner, 1974; Nosofsky, 1987; Shepard, 1987; Burns and Shepp, 1988; Nosofsky and Palmeri, 1996; Fifić et al., 2008; Little et al., 2013) and pitch and loudness for auditory stimuli (Grau and Kemler-Nelson, 1988). Though these dimensions meet several empirical criteria for integrality (defined further below), there is also a sense in which these dimensions are easily used to form a mental representation of the stimuli; that is, given a set of stimuli which vary in brightness and saturation, individuals are likely to form a psychological representation of the stimuli using dimensions which correspond to brightness and saturation. Consequently, these dimensions are *psychologically privileged* and fall short of Grau and Kemler-Nelson's (1988) notion of the “extreme-end” of integrality, where the individual dimensions are unable to be accessed at all.

More recently, Goldstone and Steyvers (2001; see also Gureckis and Goldstone, 2008; Hendrickson et al., 2010; Folstein et al., 2012; Jones and Goldstone, 2013) have utilized a set of morph dimensions which are thought to have no perceivable dimensional structure yet still meet the empirical criteria for integrality; consequently, these arbitrarily-defined morph stimuli may fulfill Grau and Kemler-Nelson's (1988) notion of an “extreme” integral stimulus. This renders these morphs useful for studying the difference between integral and separable dimensions. In this paper, we test whether these arbitrarily-defined morph dimensions demonstrate evidence of integrality in a task which goes beyond the classic converging operations by utilizing not only mean response time (RT) and choice comparisons, but also analysis of the full RT distributions and the time course of information processing. Our measure thus provides a more nuanced understanding of integrality than previous empirical criteria.

### Converging empirical operations for integrality

There are a number of converging operations suggesting that integral dimensions are processed differently from separable dimensions (Garner, 1974):

1. The distances between stimuli derived from proximity estimates (e.g., similarity ratings, identification confusions and so on) using multidimensional scaling (MDS) are better described by an Euclidean distance metric if the dimensions are integral but by a city-block distance metric if the dimensions are separable (Attneave, 1950; Torgenson, 1958; Shepard, 1964, 1987; Nosofsky, 1992).
2. People tend to sort integral-dimensioned stimuli based on overall similarity but separable-dimensioned stimuli based on individual dimensions (Imai and Garner, 1968; Handel and Imai, 1972; Garner, 1974).
3. Learning to attend to important attributes takes place more efficiently for separable-dimensioned stimuli (Shepard et al., 1963; Posner, 1964; Nosofsky, 1986) than for integral-dimensioned stimuli (Shepard and Chang, 1963; Nosofsky, 1987; Nosofsky and Palmeri, 1996).
4. Integral dimensions, but not separable dimensions, tend to interfere with each other if one of the dimensions must be ignored, but tend to facilitate one another if the dimensions are varied in a correlated manner (Lockhead, 1966; Egeth, 1967; Garner, 1969; Garner and Felfoldy, 1970; Biederman and Checkosky, 1970; Garner, 1974).

Each of these operations suggests that integral dimensions are processed as an entire object (Lockhead, 1966, 1972), but separable dimensions are processed as independent, component parts of an object.

Despite this wealth of converging operations, Cheng and Pachella (1984) argue that integrality may be an artifact of testing perceptual dimensions which do not correspond to an observer's psychological representation. For example, results showing a failure of converging operations (e.g., an interference effect between purported integral dimensions but no facilitation effect, (Garner, 1974; see also Biederman and Checkosky, 1970; Levy and Haggbloom, 1971; Gottwald and Garner, 1975; Pomerantz and Sager, 1975; Smith and Kemler, 1978) reduce the “explanatory power” of the concept of integrality (Cheng and Pachella, 1984, p. 283). In order to conclusively demonstrate integrality, Cheng and Pachella (see also Grau and Kemler-Nelson, 1988) argue that one must demonstrate that the experimenter-defined and participant-defined dimensions are commensurate *and* that the dimensions still satisfied the empirical criteria for integrality. Obviously, this presents a problem for empirically justifying the integrality of dimensions at the extreme-end of integrality which are meant to be without perceivable dimensional structure.

### Arbitrary dimensions and integrality

**Figure 1:**
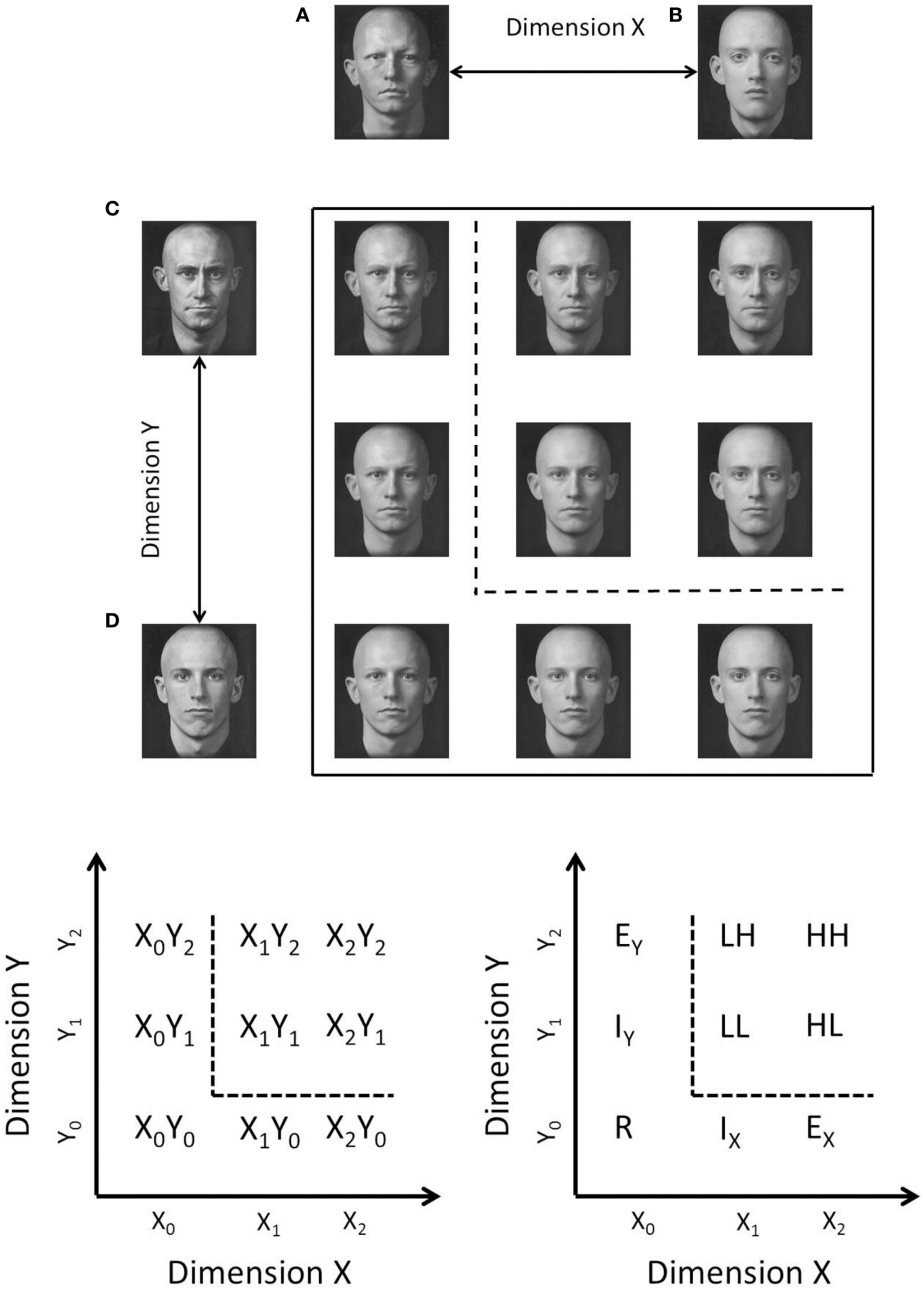
**Top:** Example of morph stimuli for Experiment 1. Each of the dimensions are created by morphing between two base faces. Each morph stimulus is a proportional mixture of all four base faces. **Bottom**: Schematic illustration of category space indicating the nomenclature used in the text. Stimuli which lie above and to the right of the decision boundary (dotted line), belong to the *target* category (category A), stimuli which low below and to the left of the decision boundary belong to the *contrast* category (category B). Stimuli in the target category are referred to by their salience which can be high (H) or low (L) depending on whether an item is far from or close to the category boundary, respectively. Contrast category items are referred to as internal (I), external (E), and redundant (R) depending on their positions in the stimulus space.

One possible set of dimensions that might satisfy the criteria of being both integral and having no identifiable dimensional structure, are the factorially-generated morph dimensions shown in Figure 1 (top panel). These stimuli are created by morphing together four base faces (e.g., Goldstone and Steyvers, 2001). The morphed stimuli vary on two dimensions, with each of these dimensions representing the transition between two of the base faces (faces A–D in Figure 1). Hence, each stimulus can be defined by its proportional value on each of the morph dimensions, but the morph dimensions are very difficult to analyze independently. The dimensions are termed arbitrary because, although each stimulus varies systematically along two face morph axes, the face morph axes do not correspond to any naturally interpretable dimensions.

Goldstone and Steyvers (2001) showed that the morph dimensions demonstrated an interference effect in the filtration condition of the Garner (1974) speeded classification task, supporting the claim that the dimensions are processed in an integral fashion. Furthermore, Folstein et al. (2012) found that there was no advantage for learning an orthogonal boundary compared to a diagonal boundary in a factorially-generated morph space such as the space shown in Figure 1 (although it is important to note that Folstein et al., used morph cars and not morphed faces). Taken together these results indicate the arbitrary morph dimensions seem to fulfill Grau and Kemler-Nelson's (1988) criteria for the extreme-end of integrality.

Despite the large number of converging operations to identify integrality, we argue that these operations are, in fact, somewhat equivocal with regard to the actual theoretical mechanism underlying the processing of integral dimensions. For example, there have been suggestions that integrality is a continuum from completely integral to completely separable (Torgenson, 1958; Shepard, 1964; Lockhead, 1972; Garner, 1974; Foard and Kemler, 1984; Grau and Kemler-Nelson, 1988; Melara and Marks, 1990) and that separable stimuli, with practice, may become integral over time (Ashby and Maddox, 1991; Goldstone, 2000; Blaha et al., 2009). Consequently, it is unclear whether integral dimensions are always processed in a consistent fashion, especially for those dimensions which, unlike brightness and saturation or pitch and loudness, may not involve “a positive correlation between the ranges of variation of stimuli associated with important consequences” in the environment (Shepard, 1991, p. 68). Indeed, many purportedly integral dimensions are not perfectly described by a Euclidean metric, but instead by a metric somewhere in-between city-block and Euclidean (Grau and Kemler-Nelson, 1988). Hence, the converging operations typically used to identify integrality do not always converge.

Furthermore, some converging operations, such as finding slower RTs in Garner's (1974) classic filtration task when compared to the corresponding control task, are open to multiple interpretations about the underlying processing architecture. For instance, in a filtration task, the number of stimuli is increased from two to four stimuli compared to the control condition. Like the control task, only one of the dimensions is relevant for classification, and the increased RT in the filtration task compared to the control task is taken as evidence that the variation on the irrelevant dimension interferes with selective attention to the relevant dimension. Such a result is used to diagnose integrality. However, rather than reflecting interference due to irrelevant variation, the increase in RT in the filtration task might simply reflect increased confusability due to the increased number of stimuli (Maddox, 1992). Indeed, increased RTs in a filtration task have been reported for stimuli that appear to be nominally separable (Shepp, 1989).

Determining whether the arbitrary morph dimensions are, in fact, processed coactively is a fundamental question, as a number of important learning results are predicated on this assumption (e.g., Goldstone and Steyvers, 2001; Gureckis and Goldstone, 2008; Hendrickson et al., 2010; Jones and Goldstone, 2013). For example, Goldstone and Steyvers (2001) trained participants to categorize face morphs using a single orthogonal category boundary; then in a second phase, transferred participants to a new boundary which was either a 90° or 45° rotation of the originally trained boundary. Participants were able to perform more accurately with the new 90° boundary than with the 45° boundary suggesting that the initially integral morph dimensions were differentiated into two orthogonal dimensions which mapped directly onto the dimensions used to create the stimuli. Although, the morph dimensions were not confirmed to be processed separably (e.g., using a Garner interference task) after training, better performance with the 90° boundary rotation than the 45° rotation suggests that the dimensions are “psychologically privileged” after training. This effect provides strong empirical evidence that learning changes perception by creating a featural or dimensional vocabulary which perceptual processes can use for future learning and decision making (Goldstone, 1998; Goldstone et al., 2000, 2008). The emergence of psychologically privileged dimensions, termed *differentiation*, has been suggested as one of the key perceptual changes underlying human development from infancy (Smith, 1989; Goldstone et al., 2011) and the development of expertise (Burns and Shepp, 1988).

This finding is somewhat controversial as other researchers have found that differentiation does not occur with other integral dimensioned stimuli (e.g., “blobs” created via the convolution of sine waves in polar coordinates varying in amplitude and frequency; Op de Beeck et al., 2003) or even other morph dimensions created using a different morphing technique (i.e., by blending four base stimuli rather than factorially combining the base stimuli as in Figure 1; see Folstein et al., 2012, for a detailed explanation of the difference). By contrast, Hockema et al. (2005) found that differentiation did occur for blob stimuli if an adaptive learning procedure, which started with categorization of the easiest items and increased the difficulty of the task by gradually moving the selection of items closer to the category boundary, was used.

In this paper, we investigate whether the morph stimuli used to demonstrate differentiation (Goldstone and Steyvers, 2001; Folstein et al., 2012) are initially processed in an integral fashion by examining a more theoretically motivated test of integrality than previously used for these stimuli. We draw on two theoretical frameworks for understanding integrality. The first, General Recognition Theory (GRT; Ashby and Townsend, 1986) grew out of the signal detection theory tradition (Green and Swets, 1966) but allowed for rigorous theoretical definition of several empirically defined notions of independence and separability (both perceptual and decisional). The second, logical rule models of categorization (Fifić et al., 2010), utilizes the representational concepts from GRT but combines these representations with processing assumptions based on sequential sampling models (Ratcliff, 1978; Busemeyer, 1985) and information processing approaches to response time (Kantowitz, 1974; Townsend and Ashby, 1983; Townsend, 1984). A further aim of this paper is to investigate the combination of assumptions necessary for explaining an individual's categorization decisions using these face morph stimuli.

### Theoretical frameworks for understanding separability and integrality

#### General recognition theory

General Recognition Theory (Ashby and Townsend, 1986) is a multivariate generalization of signal detection theory (Green and Swets, 1966). In this framework, each stimulus is represented by a distribution, often a bivariate or multivariate normal distribution, capturing the mean location of the stimulus in a multidimensional perceptual space as well as the perceptual variability associated with that stimulus. A theory of categorization decisions is made possible in this framework by assuming that a decision boundary is established in the category space (Ashby and Gott, 1988) and integrating the perceptual distribution in each category region. This value provides the probability with which a particular categorization decision is made given a particular stimulus.

**Figure 2:**
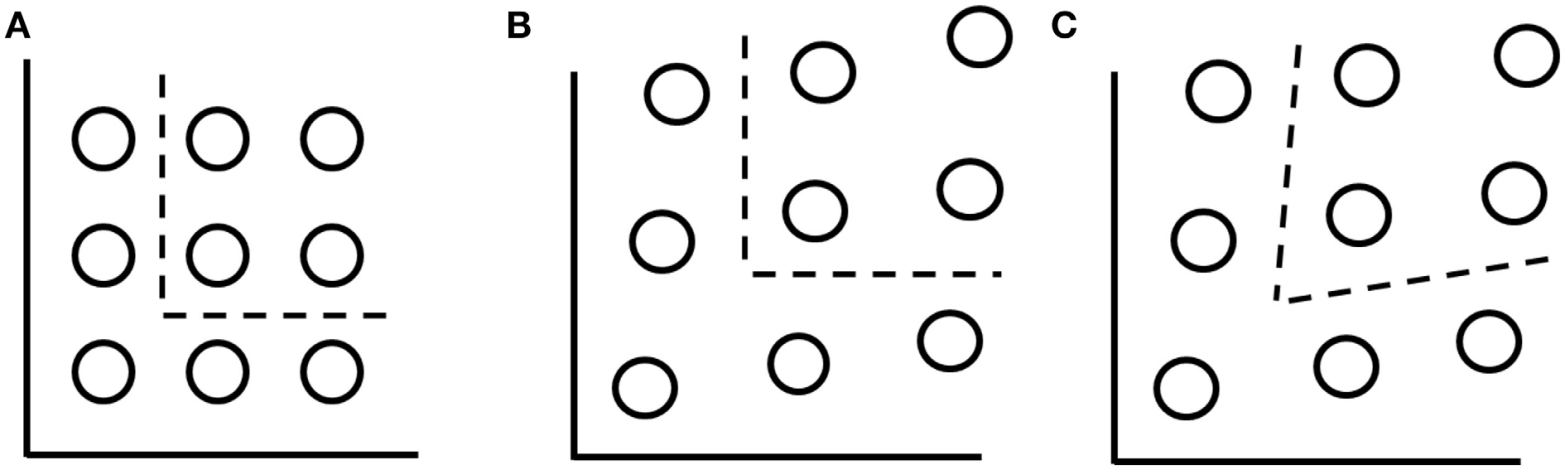
**(A)** Isoprobability contours when perceptual separability and decisional separability hold. **(B)** Isoprobability contours when there is a violation of perceptual separability due to mean shift integrality but decisional separability holds. **(C)** Isoprobability contours with mean shift integrality and an optimal decision bounds.

GRT provides a theoretical unification of differing ideas about *perceptual independence*, *perceptual separability* and *decisional separability*. For example, the category space shown in Figure 2A (GRT PS + DS) shows the isoprobability contours for nine two-dimensional stimuli. The isoprobability contours can be thought to represent a top view of a slice through the bivariate normal distributions representing each stimulus. Note that the distributions are circular representing the idea that there is no statistical correlation between the perceived values of the dimensions. This absence of correlation is termed *perceptual independence* and is a construct which refers to a single stimulus.

By contrast, separability and integrality are constructs which refer to collections of stimuli. To explain, *perceptual separability* occurs when the mean locations, and variability, of the stimuli are aligned along a dimension making it possible to represent the collection of the stimuli by the same marginal distribution along that dimension. Note that perceptual separability can occur with or without perceptual independence. A violation of perceptual separability occurs if the perceptual effect of one dimension is affected by the level of another dimensions. Although there are many ways in which this can occur, two of these violations are through varying the means of the distributions, termed *mean shift integrality*, or by altering the variances between the stimuli, termed *variance shift integrality* (Ashby and Maddox, 1994). Figures 2B,C illustrate mean shift integrality. In contrast to variation of the stimulus characteristics, *decisional separability* refers to the alignment of decision bounds with the dimensional axes of the stimuli. When decisional separability holds, the decision bound is orthogonal to the dimensional axis to which it applies. By contrast, violations of decisional separability occur when the boundaries are not orthogonal. For instance, in Figure 2C, the placement of the decision boundaries at an optimal orientation with respect to the stimuli represents a violation of decisional separability.

These constructs are important and useful because they provide a quantitative framework which can be used to predict some of the different empirical operations which differentiate performance with integral and separable dimensions; though predicting the response time effects in, for instance, Garner's (1974) classic experiments, requires auxiliary assumptions about how RTs are generated. For instance, Maddox (1992) adopted the RT-distance hypothesis which posits that RTs are a monotonically decreasing function of the distance of a stimulus from the decision boundary (Ashby and Maddox, 1991). Within this framework, facilitation for integral dimensioned stimuli when there is correlated variation between dimensions can then be explained by assuming optimal decision boundaries. By contrast, interference effects due to irrelevant dimensional variation can be explained by an increase in perceptual variability.

Nosofsky and Palmeri (1997) examined these predictions by examining the full RT distributions from a replication of Garner's (1974) conditions. These authors argued that if perceptual variability increases with irrelevant variation, then under the RT-distance hypothesis the fastest RTs from the filtration condition should be faster than in those in a control condition (with no irrelevant variation). That is the increase in perceptual variability would mean that some proportion of the RTs would be generated when the perception of the stimulus was further from the decision boundary than in a control condition. Nosofsky and Palmeri's results, however, showed that RTs were slower overall with irrelevant variation at all quantiles of the RT distribution. This result argues against the RT-distance hypothesis (see also Nosofsky and Little, 2010). However, coupling the GRT framework with other mechanisms for generating response times, such as sequential sampling models, does not make this prediction since the integrated distribution can be thought to provide a “drift rate” which represents the evidence for which a stimulus belongs to each category (cf., Ashby, 2000; Fifić et al., 2010). Furthermore, new theoretical insight can be gained by combining GRT with mental architecture approaches to understanding when stimulus dimensions are processed independently and when they are pooled together into a single process.

In summary, in the present work, we utilize the representational assumptions defined in GRT but couple these with processing-based assumptions that allow us to predict RTs for each item in the task. This is a novel departure from GRT because it allows a theoretical definition of integrality which is not based on the representation of the stimulus dimensions but on how those dimensions are processed. In the following section, we present coactivity (i.e., the pooling of information from all stimulus dimensions into a common processing channel) as a plausible theoretical definition of how integral dimensions are processed.

#### Coactivity as a theoretical definition of integrality

A novel, theoretically-driven definition of integrality can be achieved by directly contrasting the information processing of multidimensional stimuli. In particular, by using factorial experiments and analyzing full RT distributions, one can differentiate between processing which analyzes each of the dimensions independently (i.e., either in serial or in parallel) and processing which pools the dimensions together into a single processing channel (hereafter, termed *coactive* processing; Townsend and Nozawa, 1995; Townsend and Wenger, 2004). Independent channel processing and coactive processing provide a novel theoretical distinction between separability and integrality that coheres with the traditional definitions of these concepts that emphasize analytic vs. non-analytic or holistic processing.

Using a combination of non-parametric analyses and parametric response time models, Little et al. (2013 see also Fifić et al., 2008; Fifić and Townsend, 2010; Little et al., 2011) demonstrated that integral dimensions of brightness and saturation are pooled into a single, coactive processing channel, but separable dimensions, such as brightness and size, are processed independently and in multiple channels. In this paper, we test whether the arbitrarily-defined face morph dimensions also demonstrate coactivity. Before turning to our experimental results, we first briefly introduce our methodology, the logical-rule models framework, which allows identification of independent channel and coactive processing, and in turn, we describe how our experiment implements this methodology.

#### Logical-rule model framework

The logical rule-based models (Fifić et al., 2010) synthesize the representational assumptions of GRT and decision-bound theory (Ashby and Townsend, 1986; Ashby and Gott, 1988), along with sequential sampling (e.g., random walk models; Ratcliff, 1978; Townsend and Ashby, 1983; Busemeyer, 1985; Luce, 1986; Link, 1992; Ratcliff and Rouder, 1998) and mental architecture frameworks (e.g., serial vs. parallel; Sternberg, 1969; Kantowitz, 1974; Townsend, 1984; Schweickert, 1992). The models are best explained with reference to the stimulus space shown in Figure 1. In this space, nine face-morph stimuli are created by orthogonally combining two dimensions, each varying in three levels.

The four stimuli in the upper right quadrant, which are assigned to the *target* category, Category A, factorially combine an easy or *high discriminability* (H) boundary decision and a difficult or *low discriminability* (L) boundary decision across two dimensions; hence, the four target category stimuli are referred to as LL, LH, HL, and HH. The target category is defined by a conjunctive rule; that is, a stimulus must have a value on dimension X greater than the vertical category boundary *and* a value on dimension Y greater than the horizontal boundary to belong to the target category. Because the stimuli in the target category must satisfy both rules, the dimensions of these stimuli must be processed *exhaustively* (i.e., both dimensions must be processed before a target category decision can be made).

**Figure 3:**
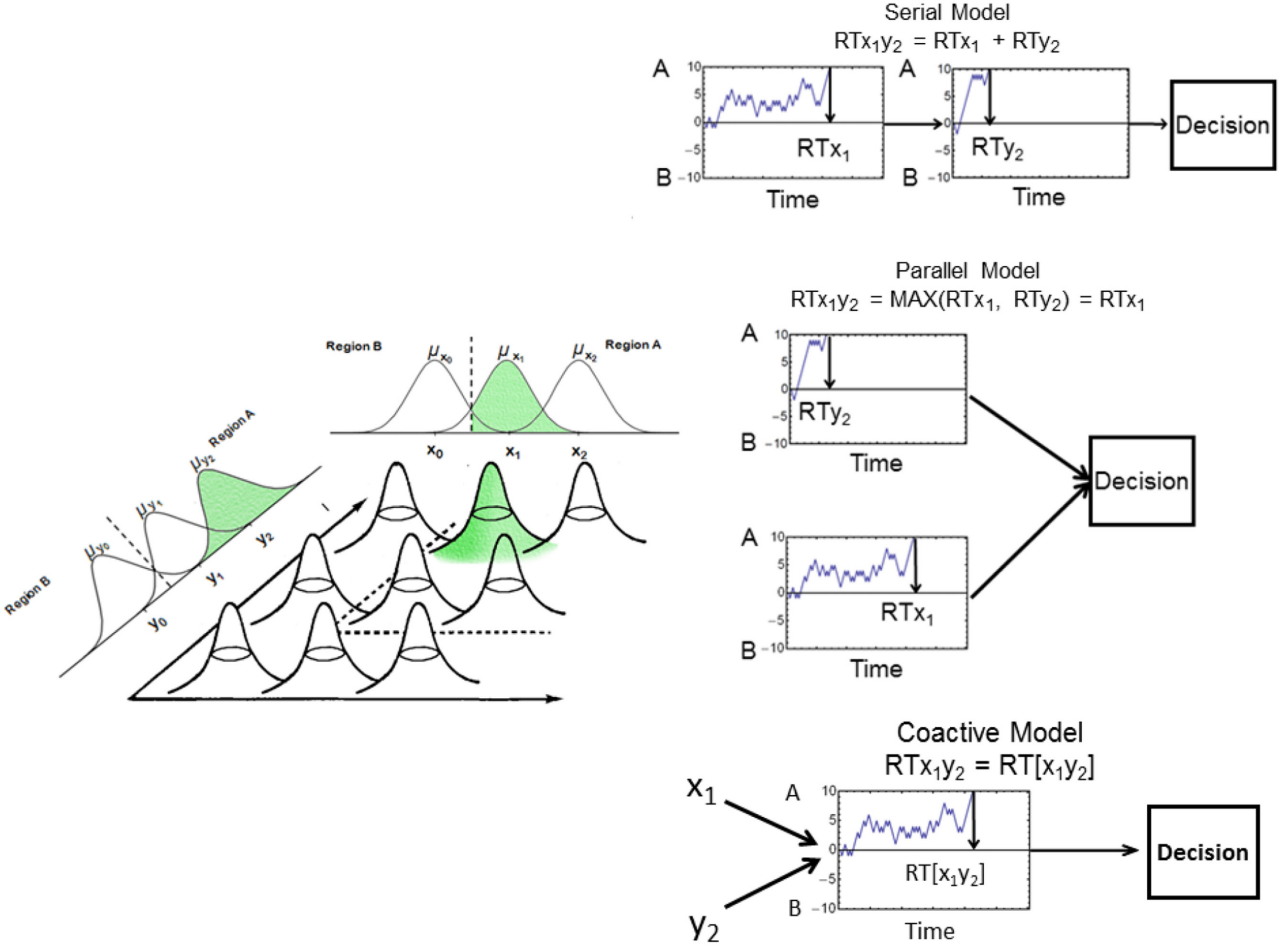
**Illustration of the random-walk process. Left**: Each stimulus is represented by a bivariate normal distribution. The dotted line represents the decision boundary. **Right**: Example of the serial (top), parallel (middle), and coactive (bottom) processing models. The serial and parallel models are driven by samples from the marginal stimulus distributions; the coactive model is driven by samples from the bivariate stimulus distribution. See text for more details.

Like GRT, the logical rule-based models (Fifić et al., 2010) assume that the perception of each stimulus dimension is represented by a normal distribution of perceptual effects. In order to make a decision, evidence is sampled from these distributions and used to drive a random walk process (see Figure 3). More specifically, following decision-bound theory (Ashby and Townsend, 1986; Ashby and Gott, 1988), observers are assumed to establish a decision boundary (represented by the dashed line in Figure 3) to separate Category A and Category B. In order to make a category decision the observer samples from the stimulus distribution using a random walk process. A sample from Category A, for example, will lead to a step toward the criterion +A. This process of evidence accumulation continues until a criterion is reached. The logical-rule models assume that the closer a stimulus is to a decision boundary in space, the more difficult it is to classify, and therefore the larger the RT.

The possible combinations of separate random-walk processes can be described using three mental architectures (i.e., serial, parallel, and coactive). For serial and parallel processes, two separate random walks occur, each driven by samples from each separate dimension. These independent random walks can occur in a serial or parallel fashion. In the case of a self-terminating stopping rule, the dimension that finishes first determines the final categorization decision and RT. In the case of an exhaustive stopping rule, however, final categorization decisions and RTs are determined by the output of both random walks.

In contrast to serial and parallel processing, coactive processing assumes that a single random walk model is driven by samples from a joint bivariate normal distribution on both dimensions X and Y. At each time step, a sample is drawn from the bivariate distribution representing the particular stimulus. If the sample falls in the Category A region, the model will take a step toward the decision criterion +A. However, if the sample falls in the Category B region, the random walk will take a step toward the decision criterion −B. This single, pooled random-walk process continues until one of the criteria is reached.

### Analysis of model predictions

As described by Fifić et al. (2010), the *double factorial* combination of the dimensional values in the target category allows us to leverage several non-parametric measures known as *Systems Factorial Technology* (SFT; Townsend and Nozawa, 1995; Townsend and Wenger, 2004) to qualitatively differentiate the candidate models. For example, the mean interaction contrast (MIC) and survivor interaction contrasts (SIC) can be used to differentiate serial, parallel, and coactive information processing architectures. These non-parametric analyses require correct stochastic ordering (i.e., *stochastic dominance*) for items in the target category. To explain, the RT for the HH face is expected to be faster than RT for the LL face since the former is further away from the category boundary than the latter. In order for the qualitative predictions to provide meaningful diagnostic information, the RTs for the HL and LH faces should be between the HH and LL faces. This ordering is reflective of the effective selective influence (Townsend and Nozawa, 1995; Heathcote et al., 2010; see also Schweickert et al., 2000; Dzhafarov, 2003; Dzhafarov et al., 2004; Dzhafarov and Gluhovsky, 2006) of each of the dimensions on the RT. Under the condition of selective influence, the MIC and SIC provide an empirically-observable, non-parametric measure which speaks directly to theoretical questions about the processing architecture and the underlying stopping rule.

Piloting of the experimental stimuli revealed that most participants demonstrated a violation of stochastic dominance, even after extended categorization training. Consequently, the current experiments will not report the SFT analyses to differentiate between information processing architectures. Instead, we will only fit RT distributions to the logical-rule models, and utilize model comparison to differentiate between mental architectures. (Further information about these analyses is available from the authors upon request).

### Processing differences for separable and integral-dimensioned stimuli

To date, a number of different dimensions and stimulus manipulations have been analyzed using this logical-rules framework. Across experiments, the largest differences in processing have been observed between separable-dimensioned and integral-dimensioned stimuli. For instance, when the stimulus dimensions were separable and located in spatially-separated locations (Fifić et al., 2010; Little et al., 2011) processing of the dimensions was best explained by a serial and self-terminating model. When separable dimensions were spatially overlapped (Little et al., 2011; Experiment 2), processing was best described as a trial-by-trial mixture of serial and parallel processing. By contrast, when the stimulus dimensions were integral (i.e., Munsell colors varying in brightness and saturation; Fifić et al., 2008; Little et al., 2013), processing conformed to the predictions of the coactive model.

To highlight the large effects of separability and integrality on processing, it is worthwhile noting that several manipulations had very little effect on processing (Fifić et al., 2010; Little et al., 2011). For instance, with separable dimensions, processing was serial regardless of whether observers were given the rule that defined the categories upfront, whether the rule had to be learned via trial-by-trial feedback, whether observers were instructed to focus on responding quickly or on responding accurately, and whether the dimensions were spatially separated or part of a single object (cf. Fifić et al., 2010; Little et al., 2011).

### Relationship to GRT's definitions of separability and integrality

In previous studies, the application of the logical rule models has always assumed perceptual independence, perceptual separability, and decisional separability. In those studies, the full RT distributions from the entire collection of stimuli from both categories could be accounted for by varying only the architecture used to determine how the information from each dimension was integrated over time. Little et al. (2013) tested whether allowing mean shift integrality and diagonal decision boundaries would allow, for instance, a parallel model to mimic a coactive model when fitting the integral dimensioned data. In that analysis, mean shift integrality was introduced by shifting the means of the stimuli so that they lied on a tilted parallelogram rather than a square grid. Even with this systematic violation of perceptual separability, neither a serial model nor a parallel model could mimic the coactive model's predictions.

Nonetheless, it is reasonable that less systematic shifts in stimulus location might require allowing for violations of perceptual separability and decisional separability. In the following, we analyze the RT distributions from individual categorization responses using the face morph stimuli shown in Figure 1. In analyzing this data, we fit several models which allow for differences in processing architecture (serial, parallel, and coactive), stopping rule (self-terminating vs. exhaustive) as well as violations of perceptual and decisional separability. To limit the scope of the project, in addition to the categorization data, we also collected similarity ratings for each pair of stimuli which we use to derive an MDS solution that can inform whether perceptual separability holds or is violated. For example, by constraining the MDS solution to lie on a grid (e.g., Borg and Groenen, 2005) we enforce perceptual separability, but by allowing the mean locations of the stimuli to vary, we capture any violations of perceptual separability.^1^ The MDS solutions also act as a further independent empirical assessment of stimulus integrality since we can also test whether the scaling solution is better fit using a city-block or Euclidean metric (Attneave, 1950; Torgenson, 1958; Shepard, 1964, 1987; Nosofsky, 1992). Our approach therefore combines three major theoretical approaches to understanding separability and integrality: GRT, MDS and the logical-rule modeling framework.

Finally, we also assumed that the decision boundaries might be either orthogonal to the decision axes or rotated to capture the optimal discrimination between stimuli from the target and contrast categories. Consequently, for each of the mental architectures, we tested three different sets of the assumptions about the perceptual representation:

1. By assuming perceptual separability (represented by using stimulus coordinates found using a constrained MDS solution) and decisional separability (orthogonal decision bounds).
2. By assuming violations of perceptual separability (by using an unconstrained MDS solution) and decisional separability (orthogonal decision bounds).
3. By assuming violations of both perceptual and decisional separability (represented by using stimulus coordinates found using an unconstrained MDS solution and by allowing optimal decision boundaries).

## Experiment 1

We examined a set of purportedly integral stimuli created from arbitrary morph dimensions. By using the conjunctive category design shown in Figure 1, we test whether the morph stimuli are processed in a coactive fashion or whether the morph dimensions are better described by an independent channel processing model (i.e., parallel or serial processing). We utilized these face morphs in both an upright and inverted orientation to extend the generalizability of our basic procedure. There is a possibility that upright faces are processed holistically, whereas inverted faces are not (Yin, 1969). However, there is a dimensionality to these face morphs which is relevant for categorizing both the upright and inverted faces (i.e., unlike for, say, recognizing upright vs. inverted faces in daily life), and consequently, we do not *a priori* expect a difference between them.

### Method

#### Participants

Eight participants from the University of Melbourne community with normal or corrected-to-normal vision were randomly assigned into the *upright* condition and the *inverted* condition with four in each condition (labeled U1–U4 and I1-I4 for the upright and inverted conditions, respectively). Participants received $12 for each session plus an extra $3 bonus for accurate performance (over 90% accuracy) during categorization sessions. All procedures were approved by the University of Melbourne Human Ethics Advisory Group.

#### Apparatus and stimuli

A category space was created using a field morphing technique (Steyvers, 1999), to morph four base faces together into a two-dimensional array (i.e., each dimension was a systematic blend from one face to a second face; Figure 1), creating a 3 × 3 matrix of faces, that are composed of factorial proportions of each of the four base faces. The base faces used in this study were identical to base faces used in Goldstone and Steyvers (2001, Experiment 1; Kayser, 1984). Dimension X was formed using the morph between faces C and D and Dimension Y was formed using the morph between faces A and B (see Figure 1). Each face in the stimulus space can be defined by a factorial combination of values on Dimension X and Dimension Y. Stimuli in the inverted condition were rotated 180°, but were otherwise identical. The stimuli were presented at a monitor resolution of 1280 × 1024 and subtended a visual angle of approximately 10°. RTs for categorization sessions were collected using a calibrated response time box (Li et al., 2010).

### Procedure

#### Categorization

Each participant completed a series of 1-h sessions on consecutive or near consecutive days for five sessions. At the beginning of each session, participants were shown experimental instructions, including example stimuli relevant to their condition (i.e., upright or inverted faces).

Each session consisted of 819 trials (9 practice trials and 810 experimental trials, divided into 9 blocks of 90 trials). Although each stimulus was presented 10 times during each block, presentation of stimuli was randomized. In between each block, participants were instructed to take a short break and were given feedback on their percentage accuracy. Participants advanced to the next block by pressing any button on the RT box. During each trial a fixation cross was presented for 1170 ms. After 1070 ms a warning tone was presented for 700 ms. A face was then presented and the participant was required to decide whether the face belonged to Category A or Category B. Faces were presented until a response was made. Feedback was provided only after incorrect responses; feedback “too slow” was provided for RTs greater than 5000 ms.

#### Similarity ratings

We ran a similarity rating study using Amazon Mechanical Turk to obtain similarity ratings for the faces shown in Figure 1. In two conditions, participants rated the similarity of the stimuli in either the upright or inverted condition of Experiment 1. A single Human Intelligence Task (HIT) was created on Amazon Mechanical Turk with 40 assignments. We restricted access to the HIT by requiring users to have at least a 90% acceptance rate (i.e., 90% of a user's completed HITs were accepted by the requester), having completed at least 1000 approved HITs, and were located in the United States. Participants were paid $2.00 USD to complete the task, which took approximately 25 min to complete. Allocation of participants to conditions was random; this resulted in 20 participants in upright condition and 20 participants in the inverted condition.

On each trial, a pair of stimuli was presented in the upper-left and upper-right of the screen. Subjects rated the similarity of each pair from 1, “least similar” to 8 “most similar.” Subjects were instructed to try to use the full range of ratings, and were given examples of high, medium, and low similarity pairs using a different set of upright faces before commencing the task. For each condition, there were 36 unique pairings of the 9 stimuli. Each pair was presented six times for each subject; the order of presentation was completely randomized as was the left-right presentation of each face. The experiment was self-paced.

### Results

For the categorization task, any trials with RTs less than 200 ms or greater than 3 SDs above the mean were removed from the analysis. No trials were removed using this method. The first session was considered practice and discarded from these analyses. Mean RTs and error rates for each participant are reported in Table 1. In the upright condition, error rates across items were low; only three items showed error rates above 10% (LH and E_X_ for U2, and LH for U3). As expected the greater difficulty in processing of inverted faces resulted in higher error rates for all four participants in the inverted condition. Participants I1 and I2 showed high error rates across all items (>20%), with very poor accuracy for items HL, LH, LL, and E_X_ and E_*Y*_. Overall error rates for participants I3 and I4 were comparatively lower (12 and 16% respectively). Similar to I1 and I2, items HL, LL, E_X_, and E_Y_ were poorest for I3 and I4. All four participants showed high error rates for item LL. This is unsurprising since LL lies adjacent to both decision boundaries.

**Table 1:** **Mean RTs and error rates for each stimulus**.

**Subject**	**HH**	**HL**	**LH**	**LL**	**Ex**	**Ix**	**Ey**	**Iy**	**R**
**EXPERIMENT 1**									
**Mean RTs**									
U1	846.65	1045.90	1092.50	1083.70	749.29	757.55	1034.40	834.62	739.03
U2	726.59	824.60	928.07	762.62	779.13	781.31	946.26	787.91	707.82
U3	643.29	812.05	766.35	741.77	626.60	627.55	708.08	604.06	525.17
U4	504.02	570.28	582.97	535.11	492.51	497.92	520.67	524.22	450.07
I1	820.75	840.61	888.08	949.38	910.41	815.71	858.14	769.13	746.85
I2	764.86	851.34	878.78	924.04	1036.00	830.62	752.88	740.32	685.86
I3	1362.50	1656.30	1459.90	1847.00	1534.70	1653.50	1624.00	1528.20	1551.00
I4	978.05	1424.90	1198.40	1394.00	1292.90	1152.80	1341.50	1376.10	966.11
**Error rates**									
U1	0.03	0.08	0.06	0.23	0.10	0.01	0.16	0.00	0.00
U2	0.03	0.18	0.14	0.20	0.12	0.04	0.12	0.18	0.01
U3	0.00	0.00	0.04	0.03	0.01	0.01	0.01	0.00	0.00
U4	0.02	0.03	0.12	0.08	0.04	0.01	0.09	0.07	0.00
I1	0.05	0.06	0.21	0.17	0.03	0.00	0.10	0.30	0.03
I2	0.01	0.02	0.03	0.10	0.11	0.01	0.06	0.02	0.00
I3	0.01	0.15	0.07	0.08	0.11	0.09	0.09	0.03	0.00
I4	0.00	0.03	0.05	0.08	0.04	0.02	0.09	0.01	0.01
**EXPERIMENT 2**									
**Mean RTs**									
U5	738.05	769.17	790.8	853.55	792.6	696.99	821.56	701.51	642.98
U6	842.46	890.03	962.3	957.15	867.32	830.68	866.29	906.27	777.98
U7	577.65	641.28	711.48	782.32	653.74	635.23	677.29	637.93	549.97
U8	1105.4	1514	1662.4	1713.8	1302.1	1097	1311.1	1643	983.33
I5	861.98	1000.6	1021.1	1056.7	787.89	717.57	940.36	1048.7	813.29
I6	752.23	916.65	918.9	1202.3	998.28	737.51	1020.9	821.31	664.97
I7	917.49	1410	1183.3	1696.5	1082.7	1188.7	1235.6	1121.1	951.46
I8	829.3	1038.3	1006.8	1140.5	965.36	1038.9	1016.6	921.6	910.21
**Error rates**									
U5	0.01	0.02	0.08	0.09	0.01	0.00	0.03	0.02	0.00
U6	0.00	0.03	0.12	0.01	0.02	0.01	0.11	0.02	0.00
U7	0.01	0.08	0.12	0.03	0.02	0.10	0.07	0.03	0.00
U8	0.00	0.04	0.05	0.01	0.01	0.02	0.02	0.02	0.00
I5	0.09	0.24	0.25	0.58	0.47	0.14	0.34	0.10	0.04
I6	0.07	0.19	0.26	0.60	0.47	0.13	0.14	0.06	0.01
I7	0.01	0.12	0.08	0.29	0.13	0.18	0.18	0.07	0.03
I8	0.00	0.29	0.06	0.40	0.19	0.09	0.14	0.21	0.02

### Computational modeling

#### Multidimensional scaling of similarity ratings

We first sought to identify participants who utilized the entire rating scale as instructed; consequently, we computed the multinomial likelihood of the counts of each rating value 1 to 8 (i.e., across all pairs) assuming that responses were (a) generated uniformly for each rating value, (b) assuming that responses were sampled primarily from only one rating value and (c) assuming that responses were sampled primarily from only two rating values. That is, each of these assumptions was used to generate a prior probability of selecting each of the response options [e.g., (a) with equal probability for each response option, (b) with most of the probability on one response option, or (c) with most of the probability spread across two response options]. Using these prior probability distributions and a multinomial likelihood, we computed the posterior probability for each hypothesis given the observed distribution of counts across rating values, using Bayes' rule. We then removed any observer with a posterior probability less than 0.5 for the uniformly distributed rating hypothesis. This resulted in the removal of two participants from the upright condition and six participants from inverted condition

**Figure 4:**
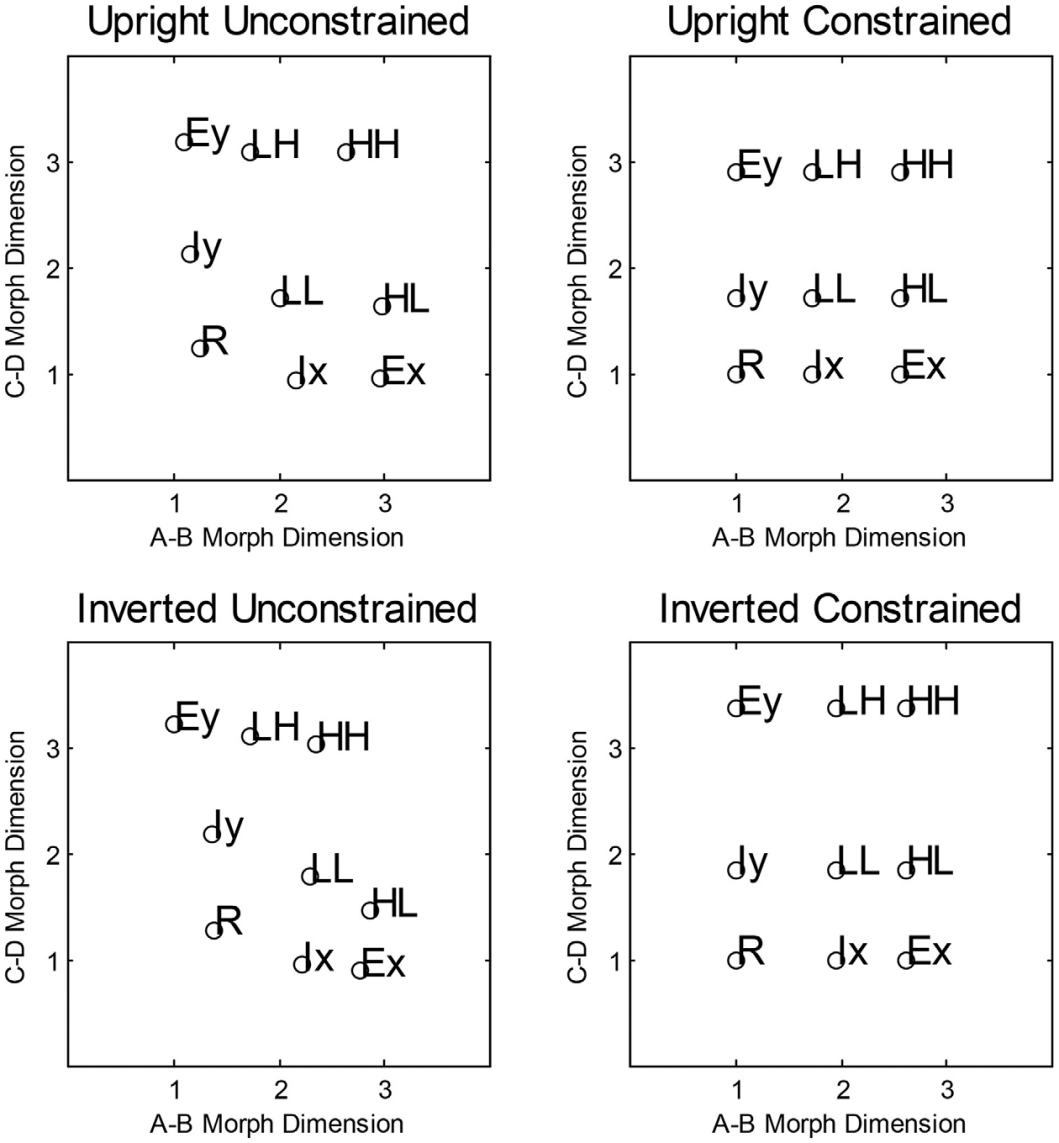
**Average multidimensional scaling solutions collected via Amazon Mechanical Turk for Experiment 1**. The **top panels** show the unconstrained and constrained scaling solutions for the Upright condition. The **lower panels** show the corresponding solutions for the Inverted condition.

We computed the averaged similarity rating for each pair of stimuli and found the two-dimensional scaling solutions for each condition. This was done by fitting the averaged ratings using a model which assumed a negative linear relationship between the predicted similarity ratings and the Euclidean distance between the estimated coordinates. To find the best fitting coordinates, we minimized the sum-of-squared deviations between the predicted and observer ratings from 100 starting points chosen to span the coordinate space. There were 20 parameters in total (the nine coordinate values, and the slope and the intercept of the negative linear distance-to-similarity function) used to fit the 36 similarity ratings. The estimated two-dimensional-scaling solution accounted for 97 and 99% of the variance in the averaged ratings for the upright and inverted conditions, respectively. To display the scaling solutions, we first performed a Procrustes rotation (Borg and Groenen, 2005) to the ideal coordinate values (see Figure 1). The rotated scaling solutions for the upright and inverted condition are shown in Figure 4. In general, both the inverted and upright scaling solutions conformed to the ideal category space outlined in Figure 1. In the upright condition, the scaling solution showed a pattern whereby the interior stimuli are positioned further from the (presumed location of) the orthogonal boundary compared to the exterior stimuli. In the inverted condition, the overall shape of the scaling solution is best described by a parallelogram. In particular, both the interior and exterior stimuli of the A-B and C-D morph dimensions appear to “slope” away from the orthogonal boundary.

For each condition, we also fitted a scaling solution that constrained each of the nine co-ordinates to a 3 × 3 grid. This model only had six free parameters and allowed only the distance between values on the A,B and C,D morph dimensions to vary. This constrained scaling solution accounted for 85 and 79% of the variance in the averaged ratings for the upright and inverted conditions, respectively. As explained above, the constrained and unconstrained scaling solutions allow for the examination of whether changing the perceptual representation affects the model fitting.

Finally, we fitted additional scaling solutions that assumed city-block distance instead of Euclidean distance between the estimated coordinates. The unconstrained model accounted for 94 and 98% of the variance in the averaged ratings for the upright and inverted conditions, respectively. In contrast, the constrained model accounted for 77 and 73% of the variance in the upright and inverted conditions. As illustrated in Table 2, the models assuming city-block distance provided worse fitting scaling solutions than the models assuming Euclidean distance. Consequently, better fitting scaling solutions with a Euclidean distance metric suggests that these face morph dimensions are integral dimensions.

**Table 2:** **Summary of the fits of the scaling models for Experiment 1 and 2**.

			**Cityblock**				**Euclidean**		
**Condition**		**Model**	**SSD**	**BIC**	**R^2^**		**SSD**	**BIC**	**R^2^**
**EXPERIMENT 1**									
	Upright	Full	4.73	−27.80	0.94		2.51	**−50.56**	0.97
		Constrained	17.25	−31.40	0.77		11.45	−46.15	0.85
	Inverted	Full	1.00	−66.97	0.98		0.59	**−85.72**	0.99
		Constrained	12.86	−24.99	0.73		9.88	−34.48	0.79
**EXPERIMENT 2**									
	U5	Full	1.06	10.26	0.82		1.21	15.01	0.79
		Constrained	2.92	−3.43	0.50		2.82	**−4.71**	0.52
	U6	Full	2.76	−15.76	0.91		3.17	−10.76	0.90
		Constrained	12.23	−12.29	0.61		9.27	**−22.28**	0.70
	U7	Full	6.52	−28.17	0.94		4.44	−41.98	0.96
		Constrained	21.38	−35.57	0.80		10.10	**−62.56**	0.90
	U8	Full	4.88	−31.62	0.94		3.27	−45.99	0.96
		Constrained	21.04	−29.18	0.76		12.07	**−49.20**	0.86
	I5	Full	1.37	**−82.87**	0.99		1.55	−78.31	0.98
		Constrained	11.38	−56.72	0.89		9.65	−62.65	0.90
	I6	Full	4.57	−27.17	0.94		2.93	−43.22	0.96
		Constrained	13.85	−37.43	0.81		9.90	**−49.53**	0.86
	I7	Full	11.32	−21.13	0.92		8.92	−29.73	0.94
		Constrained	35.59	−30.07	0.76		28.51	**−38.04**	0.81
	I8	Full	3.94	−16.67	0.91		2.93	−27.30	0.94
		Constrained	11.83	−27.29	0.74		8.26	**−40.20**	0.82

*SSD, Sum of Squared deviations; BIC, Bayesian Information Criterion*.

*The best model for each observer is shown in bold*.

#### Model fitting

Having established the coordinate values from the scaling analysis, we then estimated, for each model, the variances of the perceptual distributions, the decision boundaries, and the random walk parameters. For simplicity, we assumed equal variance across all levels of a given dimension, but allowed for differences in the variances between dimensions. As illustrated in Figure 4, the unconstrained scaling solution for both conditions deviates greatly from the ideal 3 × 3 grid layout. Given that the logical-rule models (Fifić et al., 2010) utilize the representational assumptions of GRT (Ashby and Townsend, 1986; Ashby and Gott, 1988), we can use the GRT framework to fit models that vary in the assumption of the perceptual representation of the stimuli.

We fitted three sets of models, each set containing the five possible logical-rule models, which accounted for violations of perceptual and/or decisional separability. The first set of models allowed violations of perceptual separability but maintained the assumption of decisional separability; we label this set of models MSI and DS for *mean shift integrality* and *decisional separability*. In effect, these models were fitted using the unconstrained scaling solutions and assumed orthogonal decision bounds. The second set of models assumed both perceptual and decisional separability (hereafter, PS and DS). These models were fitted using the constrained scaling solutions. The third family of models assumed both violations of perceptual and decisional separability (hereafter, MSI and OP, because the boundaries were rotated to an optimal orientation). A diagonal decision boundary was estimated using the unconstrained scaling solution. We freely estimated for each participant and each model perceptual variances, σ _*X*_ and σ _*Y*_, and decision boundaries, D_X_ and D_Y_, for Dimensions X and Y, respectively. For the optimal decision bound models, the slope (in degrees) of the decision boundaries along the X and Y dimensions was calculated prior to model fitting. The intercepts of these bounds (called *Offset1* and *Offset2*) were estimated as free parameters and they replaced parameters *D_X_* and *D_Y_* from the previously described models. For the random walk components of the models, we freely estimated response criteria +*A* and –*B*. We also assumed an additional non-decision time (i.e., time associated with encoding and movement time) was generated from a log-normal distribution \with location, μ_*r*_, and scale, σ_*r*_ and added to the decision time generated from the random walk. We further assumed that each step in the random walk was scaled to milliseconds by a multiplicative scaling constant, *k*. Hence, each of the logical rules models has nine free parameters. The sole exception is the serial self-terminating model for which we also estimated the probability that dimension X was processed before dimension Y, *p_X_*.

We fitted the models simultaneously to the correct-RT distributions and the error rates for each item by using quantile-based maximum likelihood estimation (Heathcote et al., 2002). For each item, correct RT predictions were generated for the 10, 30, 50, 70, and 90% quantiles. We did not attempt to fit the error-RT distributions since error rates were generally low. The fit of the models to the data was given using the multinomial log-likelihood function:

$$\ln L=\sum_{i=1}^{n}\ln\;\left(N_{i}!\right)-\sum_{i=1}^{n}\sum_{j=1}^{m+1}\ln\;\left(f_{ij}!\right)\;+\sum_{i=1}^{n}\sum_{j=1}^{m+1}f_{ij}\cdot \ln\;\left(p_{ij}\right)$$

where *N_i_* is the total number of times each item *i* (*i* = 1, *n*) was presented, *f_ij_* is the frequency with which item *i* had a correct RT in the *j*th bin (*j* = 1, *m*) or was an error response (*m* + 1), and *p_ij_* is the predicted probability that each item *i* had a correct RT in the *j*th bin or was an error. We compared each model's log-likelihood adjusted for model complexity using the Bayesian information criterion (BIC; Schwarz, 1978). The complexity penalty in the BIC is based on the number of free parameters and the size of the sample as follows:

$$\text{BIC}=-2\ln L+n_{p}\ln\;\left(M\right),$$

where *n_p_* is the number of free parameters and *M* is the total number of observations in the sample. Models with smaller BIC values are preferred. Predictions were generated by simulating 10,000 RTs for each item; details of the simulation method for each model are given in Fifić et al. (2010, pp. 311–317; numerical methods for generating model predictions are given in Little, 2012). The model fits for each subject in the upright and inverted conditions are shown in Table 3 and the parameters of the best fitting model are shown in Table 4.

**Table 3:** **Model Fits to subjects in Experiment 1 (model with the lowest BIC in each set is bolded; best overall model is bolded an italics)**.

**Subject**	**Coactive**		**Parallel exhaustive**		**Parallel Self-terminating**		**Serial exhaustive**		**Serial self-terminating**	
	**-lnL**	**BIC**	**-lnL**	**BIC**	**-lnL**	**BIC**	**-lnL**	**BIC**	**-lnL**	**BIC**
**PS AND DS**										
U1	290.07	616.04	471.95	979.8	***255.26***	***546.41***	380.75	797.41	308.04	655.97
U2	437.07	910.04	448.34	932.58	**419.45**	**874.79**	489.27	1014.4	443.56	927
U3	462.32	960.55	533.06	1102	452.12	940.13	**427.55**	**891.01**	441.19	922.27
U4	314.04	663.98	442.85	921.6	**264.83**	**565.55**	384.25	804.39	341.48	722.84
I1	***231.62***	***499.14***	376.03	787.96	267.22	570.33	514.06	1064	280.39	600.67
I2	***215.65***	***467.2***	400.75	837.4	256.6	549.09	531.55	1099	253.04	545.97
I3	306.93	649.76	288.92	613.75	***258.91***	***553.72***	366.95	769.8	258.42	556.73
I4	**289.2**	**614.29**	413.1	862.1	236.11	508.11	542.26	1120.4	268.18	576.24
**MSI AND DS**										
U1	**277.82**	**591.54**	418.40	872.69	307.71	651.31	349.33	734.56	309.52	658.93
U2	**417.36**	**870.61**	486.76	1009.40	419.94	875.78	470.07	976.04	425.09	890.06
U3	***358.91***	***753.72***	579.67	1195.20	444.50	924.90	462.10	960.09	446.36	932.61
U4	***234.86***	***505.62***	387.66	811.23	263.47	562.84	396.34	828.59	344.65	729.19
I1	**291.25**	**618.41**	536.66	1109.20	435.17	906.25	639.92	1315.70	457.87	955.64
I2	**296.89**	**629.68**	616.82	1269.50	481.39	998.68	716.87	1469.60	498.94	1037.80
I3	**325.64**	**687.19**	515.23	1066.40	494.15	1024.20	561.77	1159.40	504.63	1049.20
I4	***208.42***	***452.73***	558.00	1151.90	424.44	884.77	608.31	1252.50	503.77	1047.40
**MSI AND OP**										
U1	277.46	590.82	380.43	796.76	**263.48**	**562.86**	302.37	640.64	894.47	1828.84
U2	413.13	862.16	472.74	981.38	***392.59***	***821.08***	459.87	955.64	1026.32	2092.53
U3	373.81	783.52	541.82	1119.54	**366.98**	**769.86**	369.01	773.92	1115.98	2271.84
U4	275.87	587.64	419.63	875.16	**274.94**	**585.78**	358.00	751.90	1011.85	2063.59
I1	**331.61**	**699.12**	456.14	948.18	427.00	889.90	569.69	1175.28	586.19	1212.28
I2	**444.62**	**925.14**	610.14	1256.18	605.07	1246.04	726.03	1487.96	791.77	1623.42
I3	**466.31**	**968.52**	588.70	1213.30	585.75	1207.40	727.57	1491.04	786.34	1612.56
I4	**307.18**	**650.26**	645.95	1327.80	631.75	1299.40	817.53	1670.96	808.54	1656.97

**Table 4:** **Parameters for the best fitting model for subjects in Experiment 1 and 2**.

**Subject**	**Set**	**Model**	**-lnL**	**BIC**	**Dx**	**Dy**	**σ x**	**σy**	**+A**	**+B**	**μ_r_**	**σ_r_**	**k**
**EXPERIMENT 1**															
U1	PS and DS	ParallelST	255.26	546.41	1.35	1.59	0.76	0.19	5	3	6.38	0.36	38.71
U2	MSI and OP	ParallelST	392.59	821.08	1.34	1.73	0.46	0.25	4	3	6.15	0.23	45.07
U3	MSI and DS	Coactive	358.91	753.72	1.40	1.27	0.19	0.24	3	2	5.58	0.38	99.09
U4	MSI and DS	Coactive	234.86	505.62	1.43	1.28	0.13	0.13	3	2	5.81	0.16	50.40
I1	PS and DS	Coactive	231.62	499.14	1.18	0.50	4.17	2.98	4	3	6.13	0.14	37.60
I2	PS and DS	Coactive	215.65	467.20	1.44	0.50	1.61	6.12	3	3	6.04	0.16	53.86
I3	PS and DS	ParallelST	258.91	553.72	1.47	1.51	2.27	2.78	6	7	6.87	0.49	15.68
I4	MSI and DS	Coactive	208.42	452.73	0.76	1.08	1.83	7.87	7	7	6.34	0.17	18.36
**EXPERIMENT 2**															
U5	PS and DS	Coactive	289.90	615.70	2.20	1.51	4.25	2.98	9	5	6.31	0.17	7.48
U6	PS and DS	ParallelST	253.97	543.83	2.50	2.48	2.02	0.95	3	3	6.09	0.14	56.98
U7	MSI and DS	ParallelST	244.73	525.37	1.68	1.57	1.89	1.57	8	8	5.97	0.02	9.48
U8	MSI and DS	ParallelST	338.97	713.84	1.66	1.43	1.53	1.24	6	6	5.69	0.07	48.81
I5	MSI and OP	ParallelST	304.50	644.89	1.66	1.59	2.88	2.89	5	6	6.19	0.09	18.44
I6	MSI and DS	Coactive	290.12	616.13	1.46	1.16	1.35	0.46	5	4	5.96	0.14	47.22
I7	PS and DS	ParallelST	261.63	559.17	2.50	2.50	1.98	1.37	5	5	5.93	0.06	46.72
I8	PS and DS	ParallelST	299.58	635.06	2.50	2.38	1.52	0.99	6	5	6.24	0.18	26.94

*For U2 and I5, Dx and Dy refer to Offset1 and Offest2, the slope of decision bound for dimension X and Y. The value of Offset1 and Offset2 are -2.21 and 97.45°, respectively, for U2, and -1.21 and 86.79° for I5*.

#### Upright condition

Table 3 shows the best fitting model (serial, parallel, or coactive) for each participant within each set of models. Inspection of Table 3 shows that the coactive model was the best fitting model for all participants in the models assuming MSI and DS. When both PS and DS was assumed, the parallel self-terminating was the best fitting model for three of four participants (U1, U2, and U4); the serial exhaustive model best fits U3 within this set of models. However, when MSI and OP were assumed, the parallel self-terminating model provided the best fit for all four observers in the upright condition.

**Figure 5:**
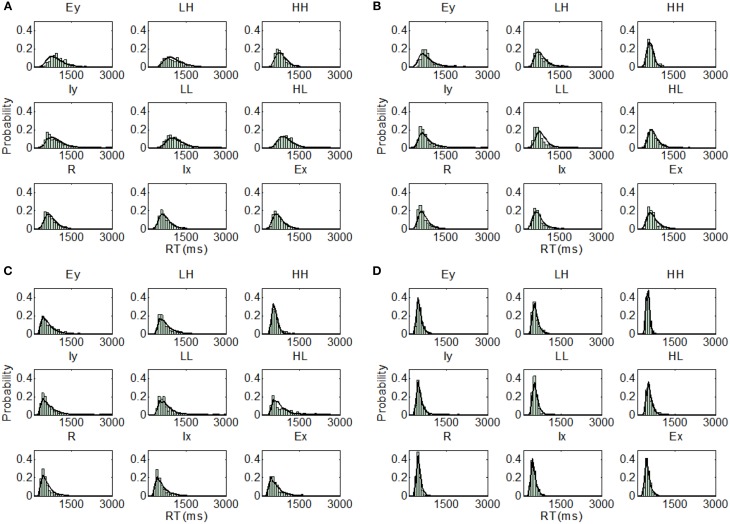
**Distribution predictions for each item using the best fitting model for each participant from Experiment 1, Upright condition (A, Subject U1—Parallel self-terminating model; B, Subject U2—Parallel self-terminating model; C, Subject U3—Coactive model; D, Subject U4—Coactive model)**.

Overall, there was a consistency of the best fitting model (parallel self-terminating or coactive) within each set of models. That is, we can rule out serial processing and, for the most part, any exhaustive processing, which accords with previous findings regarding integral dimensioned stimuli (Little et al., 2013) and stimuli with dimensions in the same spatial location (Little et al., 2011). However, in considering the best fitting model for each individual participant across all stimulus sets, there were marked individual differences. For instance, the parallel self-terminating model, was the best fitting model for participants U1 BIC = 546.51) and U2 (BIC = 821.08), and the coactive model was the best fitting model for U3 (BIC = 753.72) and U4 (BIC = 505.62). The assumption of PS also varied between these participants. The best fitting model assumes MSI and DS for U3 and U4, but the best fitting models assume PS and DS for U1, and MSI and OP for U2. The predictions of the best fitting parameters are plotted against individual RT distributions in Figure 5.

#### Inverted condition

For the inverted condition, the coactive model was the best fitting model for all participants in the two sets of models that assume perceptual integrality (regardless of decisional separability or integrality). For the set of models that assume both PS and DS, the coactive model was the best fitting model for participant I1, I2, and I4 but the parallel self-terminating model was the best model for I3.

**Figure 6:**
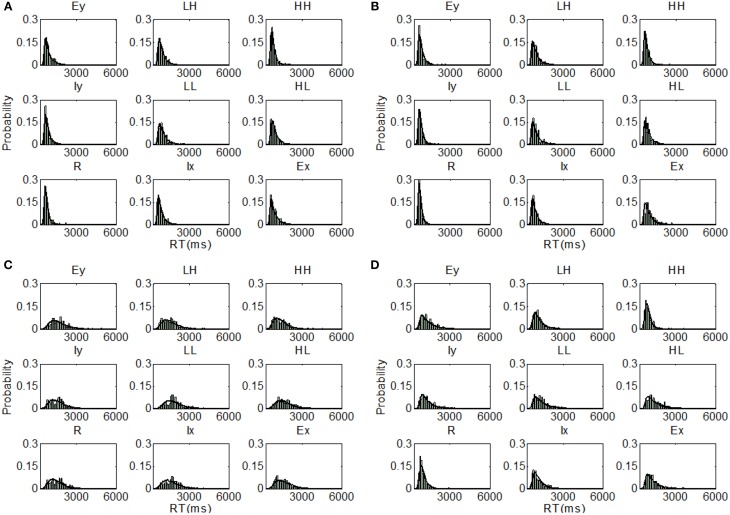
**Distribution predictions for each item using the best fitting model for each participant from Experiment 1, Inverted condition (A, Subject I1—Coactive model; B, Subject I2—Coactive model; C, Subject I3—Parallel self-terminating model; D, Subject I4—Coactive model)**.

Examining the best model across all model sets, participants I1 (BIC = 499.14) and I2 (BIC = 467.20) demonstrated coactive processing under the assumption of PS and DS. Under the same assumptions, the parallel self-terminating model was the best model for I3 (BIC = 553.72). Finally, I4 (BIC = 452.73) demonstrated coactive processing under the assumptions of MSI and DS. The predictions of the best fitting parameters are plotted against individual RT distributions in Figure 6.

In each of the logical rule models there are two key components which determine the types of predictions that are generated. The first component is the architecture of the model. The second component is the psychological representation of the stimuli, which can vary based on the nature of perceived similarity between each of the stimuli. For the current set of stimuli, we fitted a series of models by varying the assumption of perceptual and decisional separability. It is clear that changing these assumptions affects the best model for each participant. A benefit of the parametric approach taken here is that we are able to test these different assumptions in a systematic fashion.

### Discussion

Experiment 1 highlighted two important findings. First, there were individual differences in the processing of the face morph dimensions. In the general, participants in the upright and inverted conditions were best explained by either the coactive or parallel self-terminating models. Specifically, two of four participants processed the face morphs coactively in the upright condition, and three of four participants showed coactivity in the inverted condition.

Second, the best fitting model for each participant varied with changes in the perceptual representation of the stimuli. In the upright condition for example, the coactive model provided the best fit for all participants when the perceptual representation was not assumed to conform to a 3 × 3 grid-layout (see Figure 1) and when an orthogonal decision boundary was utilized. However, a parallel self-terminating model best fitted these participants when the model assumed an optimal (diagonal) category boundary. This highlights the necessity of accounting for not only architecture, but also the perceptual representation of the stimuli.

A potential caveat on this interpretation is that the scaling solution was obtained from averaged similarity ratings of online participants. Given the individual differences in processing architecture, it is highly possible that there are also individual differences in the psychological representation of the face morphs shown in Figure 1. For example, averaging the similarity data might result in greater symmetry than is observed in any of the individual participants (Ashby et al., 1994); furthermore, the results from the average data may exhibit properties which are not found in any of the individual participants. Consequently, using a single scaling solution for the computational modeling of individual participant data may mask individual differences in the MDS, and possibly also, in processing architecture. A better method would be to fit an MDS model such as INDSCAL, which allows for differential dimension weightings for each observer (Carroll and Chang, 1970). However, this would have still necessitated using an MDS solution collected from observers different from those who completed our categorization task. As an alternative, we conducted a second experiment in which in which RT distributions and scaling solutions were obtained for each participant. For this experiment, we also varied the stimulus parameters to further increase the generality of our results.

## Experiment 2

Experiment 2 replicated the upright and inverted conditions of Experiment 1 with two important alterations. First, a different stimulus space was created by swapping the positions of the two of the base faces from the set used in Experiment 1. The result of this change in base faces is that all of the stimuli except for E_Y_, LL, and E_X_ are different in Experiment 2 than in Experiment 1 (though similar because they are comprised of the same four base faces). Second, each participant completed a session of similarity ratings following their categorization sessions. Thus, participant-specific scaling solutions were used in the computational modeling.

### Method

#### Participants

Eight participants from the University of Melbourne community with normal or corrected-to-normal vision were randomly assigned into the *upright* condition and the *inverted* condition with four in each condition (labeled U5–U8 and I5-I8 for the upright and inverted conditions, respectively). Participants received $12 for each session plus an extra $3 bonus for accurate performance (over 90% accuracy) during categorization sessions.

#### Apparatus and stimuli

**Figure 7:**
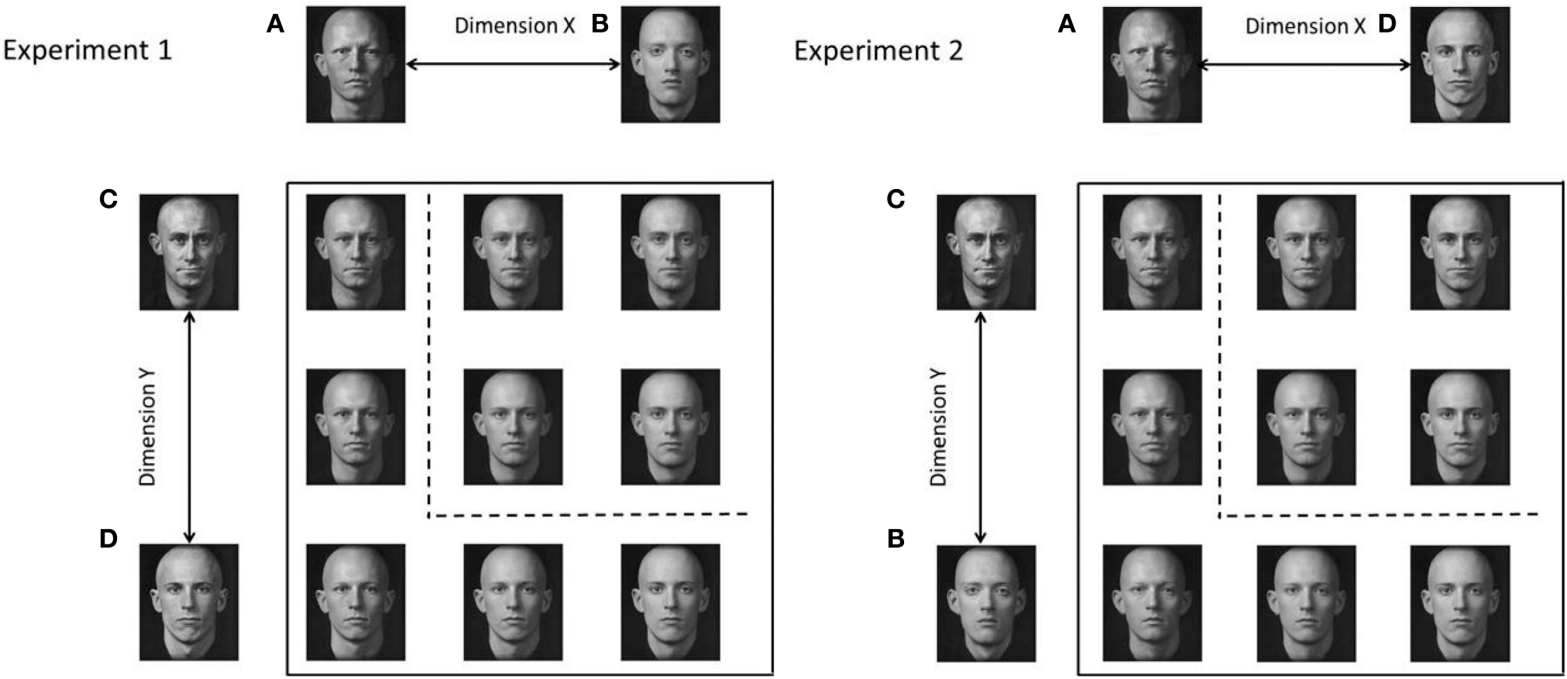
**Comparison of stimulus spaces for Experiment 1 and 2**.

The apparatus was identical to Experiment 1. The base faces used to create the stimulus space were also identical to those used in Experiment 1, however, the positions of base faces A and C were swapped. This led to a morph sequence between faces A and D, and B and C. This resulted in a different stimulus space, which was nonetheless similar as it comprised the same base faces (see Figure 7). The stimuli were presented at four degrees of visual angle.

#### Procedure

The procedure was identical to the categorization sessions of Experiment 1. Each participant completed five 1-h sessions on consecutive or near consecutive days, and only the final four sessions of categorization were used for analysis. In order to improve overall performance accuracy, participants were first shown the entire stimulus space with decision boundaries removed and were instructed take some time to study these faces to improve their performance during the experiment.

After completing the categorization sessions, participants were asked to return for a subsequent 1 h session in which they rated the similarity of the morphed faces used in the categorization task. There were 36 unique combinations of these stimuli, which were presented to participants 20 times each. On each of the 720 trials, a fixation cross was presented for 500 ms, then one of the combinations of faces was presented (i.e., two faces appeared on the screen, one face in the center of the upper right quadrant and the other in the center of the upper left quadrant of the monitor) and participants were then asked to rate the faces on the number pad using a scale of 1–8, where 1 was least similar and 8 was most similar. The presentation order of each unique pair was counterbalanced across the 20 repetitions. Comparisons were randomized for each participant. Participants in the *upright* condition made similarity judgments for upright faces, and participants in the *inverted* condition made similarity judgments for inverted faces.

### Results

For the categorization task, any trials with RTs less than 200 ms or greater than 3 SDs above the mean were removed from the analysis. This resulted in the removal of less than 1% of trials. The mean RTs and error rates are shown in Table 1, respectively. Overall, the error rates for the upright and inverted conditions were lower in Experiment 2 compared to Experiment 1, with comparable error rates between the upright and inverted conditions in Experiment 2. This shows that accuracy was approximately equal between conditions for this experiment. As seen in Experiment 1, error rates for stimulus LL in Experiment 2 were generally higher than the remaining eight stimuli.

#### Multidimensional scaling of similarity ratings

**Figure 8:**
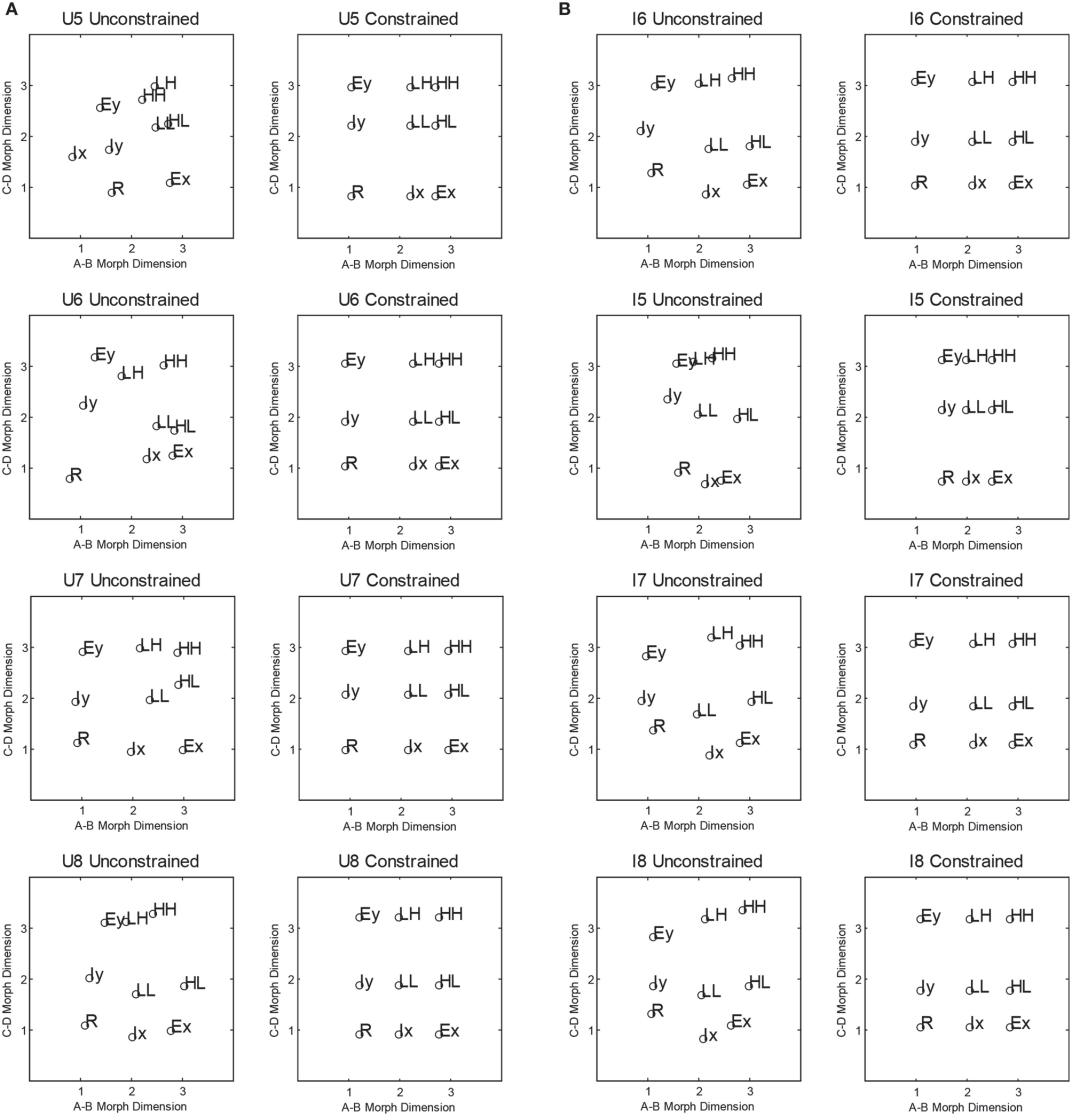
**(A,B)** shows the individual multidimensional scaling solutions for the Upright and Inverted conditions in Experiment 2, respectively.

The scaling solutions for participants in the upright and inverted conditions are presented in Figure 8. Overall, scaling solutions for each participant in the upright condition adhered to the general layout presented in Figure 1. However, participants U5 and U6 demonstrated greater deviations from the grid-layout than U7 and U8. Moreover, the unconstrained solutions revealed violations of perceptual separability for all four participants, as values on the A–D morph dimension changes with each level of the B,C morph dimension. A similar pattern of results was observed for participants in the inverted condition. Participants I6–I8 showed a perceptual representation in which items LL and I_X_ were lower on the B,C dimension than the corresponding items at that level (i.e., I_Y_ and HL, and R and E_X_). Participant I5, however, showed a pattern in which the items were more dispersed along the B,C dimension than the A–D dimension.

Similar to Experiment 1, unconstrained and constrained models assuming city-block and Euclidian distance between the estimated coordinates were fitted for each participant. A summary of the two sets of scaling solutions is provided in Table 2. For the constrained scaling solutions, models that assumed a Euclidean distance metric provided better fits of the scaling solution. A similar pattern of results was observed for the unconstrained scaling solutions. The only exception was that best fitting unconstrained solutions for subjects U5, U6, and I5 assumed city-block distance metric. Taken across all observers, the pattern suggests that these face morphs are consistent with integrality in that most observer's scaling solutions are better fit by assuming a Euclidean metric. The unconstrained model fit better but was typically less preferred based on BIC due to the larger number of parameters. Hence, based on the MDS modeling along we would conclude that for seven of our observers, there was no violation of perceptual separability. Nevertheless, we continued to utilize the unconstrained solution when fitting the different architectures to capture the assumption of MSI. As before, we also fit each of the models assuming either PS or MSI and assuming either DS or optimal category boundaries.

### Computational modeling

The model fits for each subject in the upright and inverted conditions are shown in Table 5 and the parameters of the best fitting model are shown in Table 3.

**Table 5:** **Model fits to subjects in Experiment 2 (model with the lowest BIC in each set is bolded; best overall model is bolded an italics)**.

	**Coactive**		**Parallel exhaustive**		**Parallel self-terminating**		**Serial exhaustive**		**Serial self-terminating**	
**Subject**	**-lnL**	**BIC**	**-lnL**	**BIC**	**-lnL**	**BIC**	**-lnL**	**BIC**	**-lnL**	**BIC**
**PS AND DS**										
U5	***289.90***	***615.70***	492.62	1021.14	361.32	758.54	517.10	1070.09	409.43	858.75
U6	272.71	581.32	376.66	789.23	***253.97***	***543.83***	454.94	945.78	354.61	749.11
U7	**264.74**	**565.39**	495.81	1027.51	282.97	601.85	525.06	1086.02	402.50	844.89
U8	**366.41**	**768.72**	502.23	1040.37	368.20	772.31	491.54	1018.98	458.31	956.52
I5	472.66	981.22	657.95	1351.79	**444.75**	**925.40**	744.24	1524.38	492.54	1024.97
I6	**290.34**	**616.58**	737.07	1510.05	365.19	766.28	716.51	1468.91	533.73	1107.35
I7	412.70	861.30	518.87	1073.64	***261.63***	***559.17***	514.43	1064.76	376.34	792.58
I8	372.39	780.67	368.31	772.52	***299.58***	***635.06***	410.63	857.17	353.91	747.72
**MSI AND DS**										
U5	**367.95**	**771.80**	552.57	1141.04	455.15	946.19	591.10	1210.13	447.10	934.10
U6	408.12	852.14	361.11	758.12	364.58	765.06	433.45	894.82	**340.58**	**721.05**
U7	294.90	625.71	531.11	1098.12	***244.73***	***525.37***	519.29	1066.50	286.60	613.10
U8	515.94	1067.78	538.03	1111.97	***338.97***	***713.84***	508.53	1044.99	406.77	853.43
I5	439.36	914.62	553.70	1143.29	**320.56**	**677.02**	602.31	1232.55	328.97	697.84
I6	***290.12***	***616.13***	737.84	1511.59	350.37	736.65	693.59	1415.10	468.98	977.84
I7	568.12	1172.14	610.76	1257.43	**379.92**	**795.74**	650.95	1329.82	474.17	988.22
I8	446.98	929.87	393.25	822.40	**351.66**	**739.22**	467.70	963.32	366.24	772.36
**MSI AND OP**										
U5	**409.46**	**854.82**	731.59	1499.09	1055.75	2147.40	807.25	1650.41	482.32	1004.54
U6	496.00	1027.90	490.14	1016.19	**477.51**	**990.92**	570.76	1177.42	870.17	1780.23
U7	395.85	827.60	488.78	1013.45	**309.05**	**654.01**	550.07	1136.03	1000.76	2041.40
U8	507.92	1051.75	590.52	1216.93	**356.71**	**749.31**	568.15	1172.21	1288.88	2617.65
I5	455.77	947.44	512.42	1060.74	***304.50***	***644.89***	596.91	1229.73	1202.71	2445.32
I6	720.69	1477.27	860.90	1757.70	**662.16**	**1360.23**	865.15	1766.20	1588.40	3216.69
I7	860.90	1757.70	803.03	1641.97	**801.66**	**1639.22**	837.51	1710.93	1108.51	2256.90
I8	833.26	1702.42	784.05	1603.99	**759.12**	**1554.14**	827.49	1690.87	1385.56	2811.00

#### Upright condition

Inspection of Table 5 reveals that the parallel self-terminating model was the best fitting model for three participants in the upright condition. For the set of models assuming MSI and DS, the parallel model was the best model for U7 and U8, but the coactive and serial models were the best models for U5 and U6 respectively. The parallel self-terminating model was the best fitting model for U6–U8, when assuming both MSI and OP; the coactive model was the best model for U5. For the models assuming both PS and DS, the coactive model was the best model for U5, U7, and U8, but the parallel model was the best model for U6.

**Figure 9:**
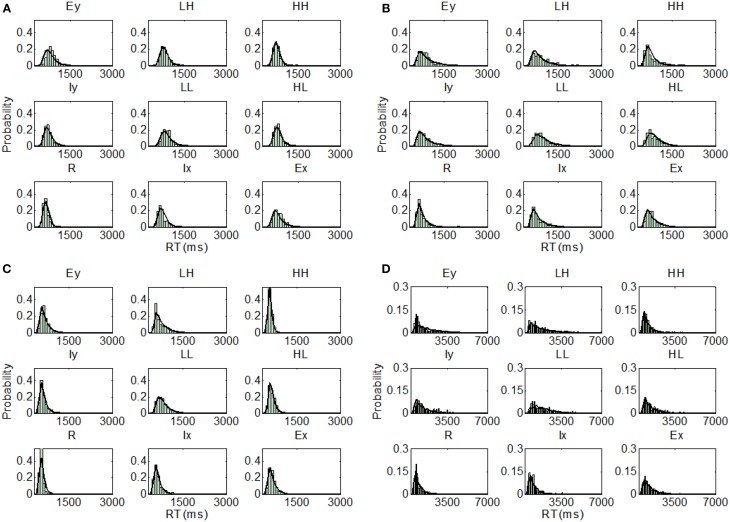
**Distribution predictions for each item using the best fitting model for each participant from Experiment 2, Upright condition (A, Subject U5—Coactive model; B, Subject U6—Parallel self-terminating model; C, Subject U7—Parallel self-terminating model; D, Subject U8—Parallel self-terminating model)**.

Individually, participant U5 demonstrated coactive processing under all three different assumptions of perceptual representation, but the model that assumes PS and DS was the overall best fitting model (BIC = 615.70). The parallel self-terminating model best fitted U6 (BIC = 543.83) with the same assumptions of perceptual representation. The parallel self-terminating model best fitted U7 (BIC = 525.37) and U8 (BIC = 713.84) under the assumption of MSI and DS. The predictions of the best fitting models are plotted against individual RT distributions in Figure 9.

#### Inverted condition

**Figure 10:**
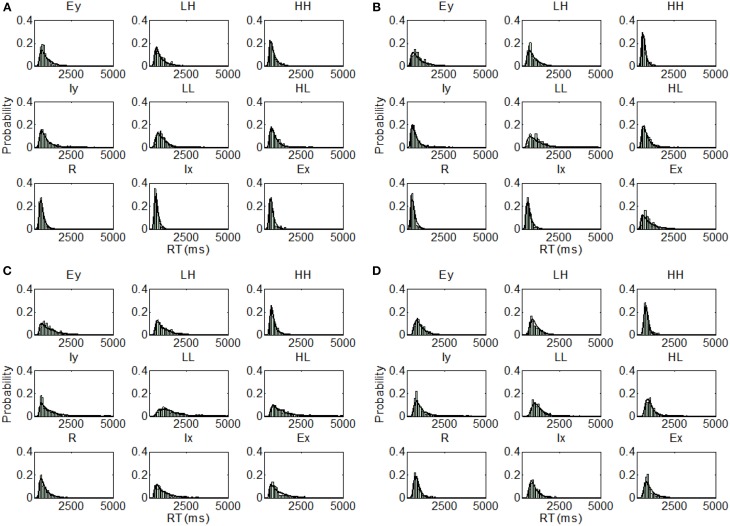
**Distribution predictions for each item using the best fitting model for each participant from Experiment 2, Inverted condition (A, Subject I5—Parallel self-terminating model; B, Subject I6—Coactive model; C, Subject I7—Parallel self-terminating model; D, Subject I8—Parallel self-terminating model)**.

The model fits of the inverted condition present a clear picture. The parallel self-terminating model best fitted the data for participants I5, I7, and I8 under all three different assumptions of perceptual representations. Participant I5 (BIC = 644.89) was best fitted with the assumption of MSI and OP, but participants I7 (BIC = 559.17) and I8 (BIC = 635.06) were best fitted with the assumption of PS and DS. For participant I6, the coactive model with the assumption of MSI and DS was the overall best fitting model (BIC = 616.13). The predictions of the best fitting parameters are plotted against individual RT distributions in Figure 10.

### Discussion

In sum, parallel self-terminating processing was observed for three of the four participants in both the upright and inverted conditions of Experiment 2. This is in contrast to Experiment 1 in which a majority of participants demonstrated coactive processing of upright and inverted face morphs dimensions. Taken together with Experiment 1, and given the small number of observers, our conclusion is that there are individual differences in the manner in which the face morph dimensions are processed. Regardless of whether the morphs are presented in an upright or inverted fashion, processing may be coactive or parallel depending on the individual observer. Similar to Experiment 1, Experiment 2 showed that changing the assumption of the underlying perceptual representations affects the best fitting model.

## General discussion

In this paper, we examined processing of purportedly integral, arbitrary morph dimensions, comparing both upright and inverted face morphs. Our primary finding was that some individuals process the dimensions in a parallel self-terminating fashion and others process the dimensions coactively for both upright and inverted face morphs.

A strength of the present study is the comparison of the model fits under different assumptions of the underlying perceptual representation. The scaling solutions from both experiments reveal deviations from the 3 × 3 grid-layout outline in Figure 1. Experiment 1 showed that the preferred model varied based on the underlying representational assumption. For example, the coactive model was the best fitting model in the upright condition for all participants when perceptual integrality and decisional separability were assumed; however, once the model assumed either optimal responding or mean shift integrality, the parallel model was superior in terms of BIC. A clear benefit of the parametric approach taken here that we are able to tease apart differences in representation from differences in architecture.

Overall, more participants used a coactive strategy in Experiment 1 compared to Experiment 2. There are two possible reasons for this difference. Firstly, participants may have perceived the face morphs differently since the visual angle and the face morph dimensions were altered between experiments (i.e., the position of two base faces were swapped). Secondly, model fitting for Experiment 1 utilized the averaged scaling solution of independent participants, but model fitting for Experiment 2 utilized individual scaling solutions after categorization training. In general, there is high variability in the perceptual representation of these face morphs between individuals and thus the average scaling solution may not have adequately represented the perceptual representation of each participant in Experiment 1.

### Implications for previous research

The finding of individual differences in processing face morph stimuli implies that previous studies employing these stimuli on the assumption that they are processed in an integral fashion need to be interpreted with caution. On the one hand, the stimuli clearly satisfy one of the empirical operational definitions of integrality in that for most observers, the best fitting scaling metric was Euclidean. On the other hand, only half of the observers required assuming a violation of perceptual separability. Furthermore, only half of the observers were best fit by a coactive processing architecture, and of those, only two observers from Experiment 2, where individual scaling solutions were used, were found to be coactive. Consequently, the evidence that the face morph stimuli provide consistent and converging evidence of coactive processing is rather weak.

In their study of perceptual differentiation, Goldstone and Steyvers (2001) found that the face morph dimensions were independently analyzable after training on a boundary orthogonal to the stimulus dimensions. Goldstone and Steyvers acknowledge the possibility that because of the grid-like arrangement of the stimuli, participants may have realized that there was a consistent dimensional structure. Indeed, in their Experiment 3, they utilized a stimulus space which did not have a grid-like structure (i.e., the face morphs were arranged in a circle), yet they still found evidence for differentiation. Consequently, it would seem prudent to limit our conclusions of individual differences to the case in which the face morphs are aligned to a grid making potentially making the dimensional structure particularly identifiable.

An alternative interpretation of our result would be to assume that differentiation is not precluded by training a category boundary on both stimulus dimensions, and that our observation that some observers processed the dimensions independent (in a parallel, self-terminating fashion) is evidence of that differentiation. In support of this idea, the MDS solution from Experiment 1, which was the only data collected prior to category learning (concerns about averaging notwithstanding; Ashby et al., 1994), is best fit by a Euclidean distance metric suggesting integrality. However, we note that a Euclidean metric was also found for most of our observers in Experiment 2 *after* extensive category learning. It is clear from the present results that individuals differ with regard to how they represent and process the face morphs used in the present study. Whether this results from a difference in the time course of differentiation and learning (i.e., across sessions) is left for future research. Nonetheless, we note that the MDS solutions found in Experiment 2 were found using data collected after extensive category learning. These solutions all indicate that a constrained solution (i.e., which exhibits perceptual separability as defined by GRT) provides a better account of the similarity data. This result is in line with the hypothesis that the stimulus dimensions were differentiated after category learning.

Finally, a further caveat on the implications of the present research is that we tested a relatively small number of individuals. This is a consequence of the experimental design which necessitates collecting large numbers of observations from each observer. Nevertheless, we can clearly rule out a large number of models including all serial models and all exhaustive models. This leaves coactivity and parallel self-termination as the remaining candidate processing models for the present face morph stimuli. That we found, essentially, the same sorts of individual differences in both experiments suggests that the individual differences are real and not due to small idiosyncratic differences between subjects.

### Implications of theoretical notions of integrality

Here we have shown that stimuli which were previously thought to be integral on the basis of one empirical test of integrality, do not necessarily meet all other tests of integrality (cf. Cheng and Pachella, 1984). The face morph dimensions used in this experiment had been previously shown to result in an interference effect when variation on an irrelevant dimension was introduced suggesting integrality. In the current study, the scaling solutions demonstrated clear violations of perceptual separability (Ashby and Townsend, 1986; Ashby and Maddox, 1991; Maddox, 1992; Maddox and Ashby, 1996) and the Euclidean metric was preferred for most observers, but for observers in Experiment 2 a constrained solution was preferred after taking the complexity of the solution into account. Taken in conjunction with the RT data, however, there was a good deal of variation in whether perceptual separability was violated or not. Little et al.'s (2013) experiments using Munsell color stimuli suggest a theoretical definition of integrality in terms of coactive processing. For the present stimuli, however, we also did not find consistently coactive processing suggesting that the face morphs used here do have some identifiable structure which can be processed in an independent fashion.

Yet, one may question why additional theoretical definitions of integrality are necessary. GRT offers a theoretical definition of perceptual representation, which rigorously defines violations of perceptual independence, perceptual separability and decisional separability, so is there any need to posit coactivity as a theoretical representation for integrality? As a background consideration, it is worthwhile to note that GRT does not predict RTs without additional mechanisms, and aside from the logical rule models presented here, only the distance-from-boundary hypothesis has been applied to explain some of the empirically observable definitions of integrality (Ashby and Maddox, 1991; Maddox, 1992). Though, as previously discussed, the distance-from-boundary hypothesis makes untenable predictions for the speed of the fastest RTs when perceptual variability is increased (Nosofsky and Palmeri, 1997). Consequently, we feel that GRT provides a representational-level theory of integrality, but does not extend adequately to understanding how integral dimensions are processed. Though we highlight recent advances in developing a non-parametric dynamic GRT, which extends the concepts defined within GRT to a class of parallel processing models (Townsend et al., 2012); these models have not yet been applied to differentiating separable and integral dimensioned stimuli. By contrast, the logical rule models approach are a process-level theory of integrality but one which offers a way to simultaneously consider both the perceptual representation and the underlying processing architecture.

There are two somewhat orthogonal ideas that might be considered when addressing the question of whether aligning integrality with coactivity is necessary. The first is that defining integrality as coactivity might confound integrality at the perceptual and decisional stages. For instance, one could imagine that perceptual separable dimensions might be pooled together at a decisional stage. While this is a conceptually possible, we do not consider this to be very plausible in the present case. This hypothesis would capture ideas present in many two-stage, salience-based models of visual search (Neisser, 1967; Wolfe et al., 1989; Wolfe, 1994; Found and Muller, 1996) that an initially independent parallel stage selects out information for later processing by an apparently coactive system. In the present case, however, the stimuli are presented until a response is generated; consequently, the early system is likely completely saturated. In this case, the GRT representations likely do not capture the early, salience-based perceptual qualities of the stimulus dimensions, but rather capture something like the relative similarity between each of the stimuli (Ashby and Perrin, 1988). Under extended display conditions, representations of the dimensions that are independent and driven by the marginal representation of the dimensions are not likely to exhibit patterns of effects which are the empirical hallmarks of integrality. The present approach allows one to test these assumptions parametrically by varying both the representation and the architecture thereby separating perceptual and decisional separability from the architecture used to generate the RTs.

A second issue arising from consideration of the mechanisms used to generate the RTs is that to the extent that integrality is aligned with the notion of holism and to what extent coactivity captures what is typically meant by that latter concept. For instance, in a task similar to the task used here, Fifić and Townsend (2010) examined the processing of secondary holistic features (e.g., the distance between the eyes or the between the lips and the nose) which are thought to be part of the underlying configural advantage underlying face perception. In that study, under conditions conducive to holistic processing, observers were found to demonstrate coactivation. Strong definitions of holistic processing seem commensurate with the theoretical notions implied by coactivity; the same is true when ideas of holistic processing are applied to dimensional integrality.

Fifić and Townsend's (2010) finding of coactivation using faces with secondary-level facial feature differences stands in contrast to the relative lack of consistent coactivation in the present experiments using face morphs. One possibility is that, like Fifić and Townsend's study, coactivation would develop over time with repeated presentation of the stimuli as the individual morph dimensions are unitized into a holistic representation. Although this is possible, it is the opposite of the direction of perceptual learning assumed by Goldstone and Steyver's (2001) in which the face morph dimensions became more separable with training. A key difference in that study was that the training only utilized discriminations along a single dimension, whereas here, both dimensions are relevant. Nonetheless, we find mixed evidence of coactivation when both dimensions are relevant. This also did not vary based on whether faces were presented in an upright or inverted fashion. We tentatively suggest that the morph dimensions we use here do not contain the sort of individual identification information that seems to drive superior face identification performance but instead contain dimensional structure which can be utilized by some observers. This clearly renders overarching inferences based on averaged data problematic. We argue that without factorial manipulations to tease apart how dimensional information is integrated for each observer, general conclusions may be misleading.

Finally, although the logical rules framework that we adopt here combines many existing approaches to studying integrality and separability, it is worth considering whether some deeper theoretical insight can be used to understand the variety of converging operations. Three converging operations are worth considering: the MDS metric (Attneave, 1950; Torgenson, 1958; Shepard, 1964, 1987; Nosofsky, 1992), the efficiency of selective attention (Nosofsky, 1987), and Garner's (1974) facilitation and interference results. When coupled with our modeling results, the finding that a Euclidean metric persists after extensive category learning suggests the distance metric is an unreliable indicator of integrality (Grau and Kemler-Nelson, 1988). This suggests that a target for future research is to determine how different processing architectures predict the types of proximity measures which are used to derive the scaling solutions. For instance, one question of interest is whether serial and parallel processing models, which compare dimensions independently necessarily always lead to solutions with city-block distance metrics. A second question is whether coactivity always leads to solutions with Euclidean distance metrics. At present, these relations are intuitive, but the strength of this relationship is unclear.

With regard to the efficiency of selective attention, in the logical rules models, there are at least two possible ways by which selective attention might influence processing. One mechanism is to increase the processing rate of attended dimensions and decrease the rate of less attended dimensions (see for example, Nosofsky and Palmeri, 1997; Ashby and Perrin, 1988). A second possibility is that selective attention might be linked to selective, fixed-order serial processing. That is, dimensions which are learned to be relevant for categorization or are more salient might be selected to be processed before (or to the exclusion of, in a self-terminating model) less relevant or less salient dimensions. In support of this idea, Lamberts (1995; see also Cohen and Nosofsky, 2003) showed that for separable-dimensioned stimuli, attributes vary in their temporal order within the decision process with more salient dimensions processed before other dimensions. These results are consistent with the logical rules account of serial processing of separable dimensioned stimuli (see Fifić et al., 2010), and by contrast, support the idea that integral dimensions should be process coactively (Little et al., 2013).

As noted in the introduction, Garner's (1974; Garner and Felfoldy, 1970) tasks do not allow one to differentiate between different processing architectures. The reason for this is that these tasks involve categorization using a single relevant dimension. Under these conditions, there is no difference in the processing rate predicted using the joint bivariate distributional representation and the marginal distribution representation. Likewise, there is only one processing channel (i.e., the relevant dimension). Hence, separable dimensions, which show no facilitation (i.e., with correlated variation) or interference (i.e., with irrelevant variation) might be processed either in a serial, a parallel, or a coactive fashion. On the other hand, the signature integral result of facilitation and interference could indicate either coactivity or some form of parallel processing. To explain, we consider coactivity to be likely for integral dimensions, but a change in architecture alone cannot predict Garner's results for integral dimensions. As discussed in Little et al. (2013, p. 817), other representational mechanisms would need to vary to predict facilitation and interference. For instance, one might expect optimal responding (i.e., a diagonal decision bound; Maddox, 1992) with correlated variation or an increase in perceptual variability (Maddox, 1992; Nosofsky and Palmeri, 1997) with irrelevant variation. However, the latter interference result could also be predicted via other mechanism; for instance, parallel processing with increased response caution could cause a slowing of RTs with irrelevant dimensional variation. We offer the method we employ in the present paper, which combines both processing and representational assumptions, as a framework for addressing these complex issues.

### Conflict of interest statement

The authors declare that the research was conducted in the absence of any commercial or financial relationships that could be construed as a potential conflict of interest.

## Footnotes

^1^We do not examine variance shift integrality (or other violations of perceptual separability) in this paper because when coupled with the decision boundary, the effect of changing the mean or changing the variance of a perceptual distribution in the logical rule models is to change the probability that the random walk takes a step up or down toward the +A or −B boundary. We considered it unlikely that we would be able to differentiate these two accounts using the present design and instead leave that for future research.
